# Supplementary material for: Tissue-specific multi-omics analysis of atrial fibrillation
Source: Nat Commun. 2022 Jan 21;13:441. doi: 10.1038/s41467-022-27953-1 (PMC8782899; doi:10.1038/s41467-022-27953-1)
Supplement: Supplementary file 1 — Supplementary information [file 41467_2022_27953_MOESM1_ESM.pdf]

SUPPLEMENTARY INFORMATION:

Tissue-specific multi-omics analysis of atrial fibrillation

Ines Assum[1,2,†], Julia Krause[3,4,†], Markus O. Scheinhardt[5], Christian Müller[3,4], Elke Hammer[6,7], Christin S. Börschel[4,8], Uwe Völker[6,7], Lenard Conradi[9], Bastiaan Geelhoed[4,8,10], Tanja Zeller[3,4,*], Renate B. Schnabel[4,8,*], Matthias Heinig[1,2,11,*]

[1]Computational Health Center, Helmholtz Zentrum München Deutsches Forschungszentrum für Gesundheit und Umwelt (GmbH), Neuherberg, Germany.
[2]Department of Informatics, Technical University Munich, München, Germany.
[3]University Center of Cardiovascular Science, University Heart and Vascular Center Hamburg, Hamburg, Germany.
[4]Partner site Hamburg/Kiel/Lübeck, DZHK (German Center for Cardiovascular Research), Hamburg, Germany.
[5]Institute of Medical Biometry and Statistics, University of Lübeck, Lübeck, Germany.
[6]Interfaculty Institute for Genetics and Functional Genomics, University Medicine Greifswald, Greifswald, Germany.
[7]Partner site Greifswald, DZHK (German Center for Cardiovascular Research), Greifswald, Germany.
[8]Department of Cardiology, University Heart and Vascular Center Hamburg, Hamburg, Germany.
[9]Department of Cardiovascular Surgery, University Heart and Vascular Center Hamburg, Hamburg, Germany.
[10]Department of Cardiology, University of Groningen, University Medical Center Groningen, Groningen, Netherlands
[11]Partner site Munich, DZHK (German Center for Cardiovascular Research), Munich, Germany.

[†],* These authors contributed equally.
Correspondence to Matthias Heinig matthias.heinig@helmholtz-muenchen.de or Renate B. Schnabel r.schnabel@uke.de.

Supplementary Figures and Figure Legends

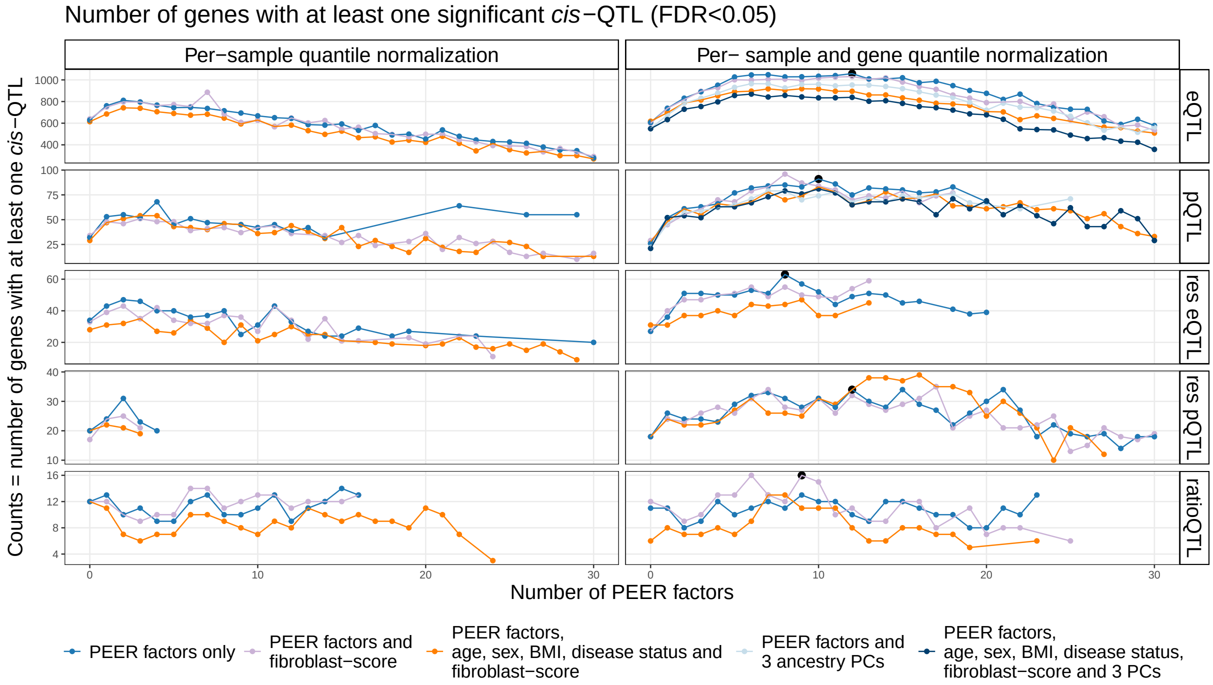

Figure 1: *Cis*-QTL analysis results for different covariate sets and number of PEER factors. PEER analysis was performed to account for unknown variation in the data. Displayed are the number of genes with at least one QTL variant with a FDR<0.05 for different combinations of normalization, number of PEER factors used in the regression and additional covariates. Black dots mark the chosen number of PEER factors and covariates as the maximal number of discovered QTL genes at a FDR<0.05.

QTL, quantitative trait loci; PEER, probabilistic estimation of expression residuals; FDR, false discovery rate; eQTL, expression quantitative trait loci; pQTL, protein quantitative trait loci; res eQTL, expression residual quantitative trait loci; res pQTL, protein residual quantitative trait loci; ratioQTL, ratio quantitative trait loci; Source data are provided as a Source Data file.

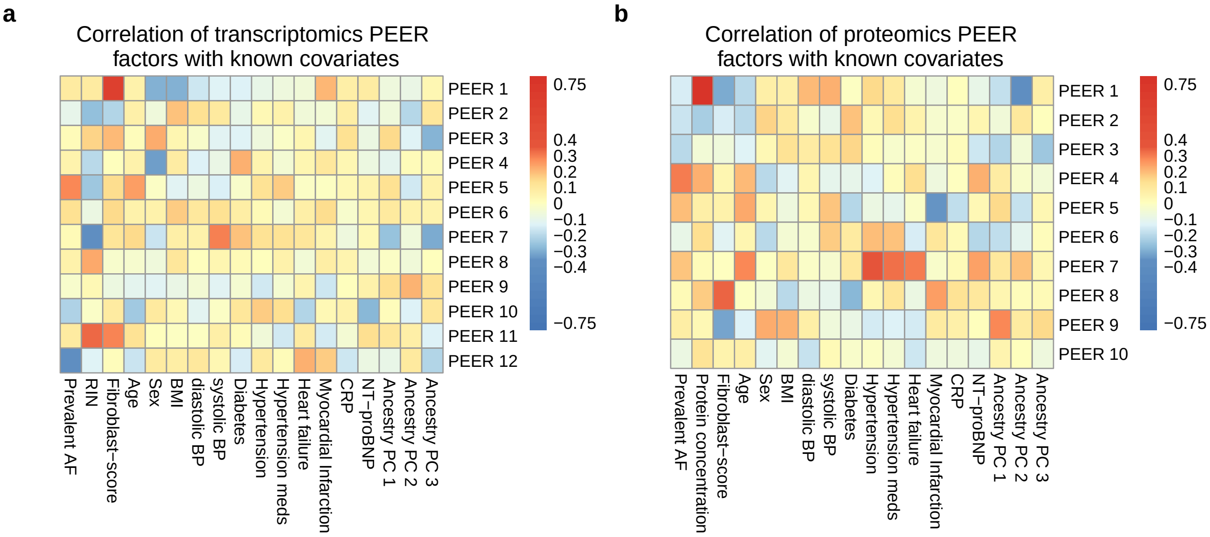

Figure 2: Correlation of PEER factors with common risk factors of AF and technical covariates. Pearson correlation of different PEER factors and known risk factors or technical covariates.
a: Transcriptomics analysis: Fibroblast-score and RIN-score highly correlate with PEER factors used in the final *cis*-eQTL analysis.
b: Proteomics analysis: Fibroblast-score and original sample protein concentration highly correlate with PEER factors used in the final *cis*-pQTL analysis.
PEER, probabilistic estimation of expression residuals; QTL, quantitative trait loci; AF, (prevalent) atrial fibrillation; RIN, RNA integrity number; BMI, body mass index; diasBP, diastolic blood pressure; sysBP, systolic blood pressure; HF, heart failure; MI, myocardial infarction; CRP, C-reactive protein; NT-proBNP, N-terminal prohormone of brain natriuretic peptide; Source data are provided as a Source Data file.

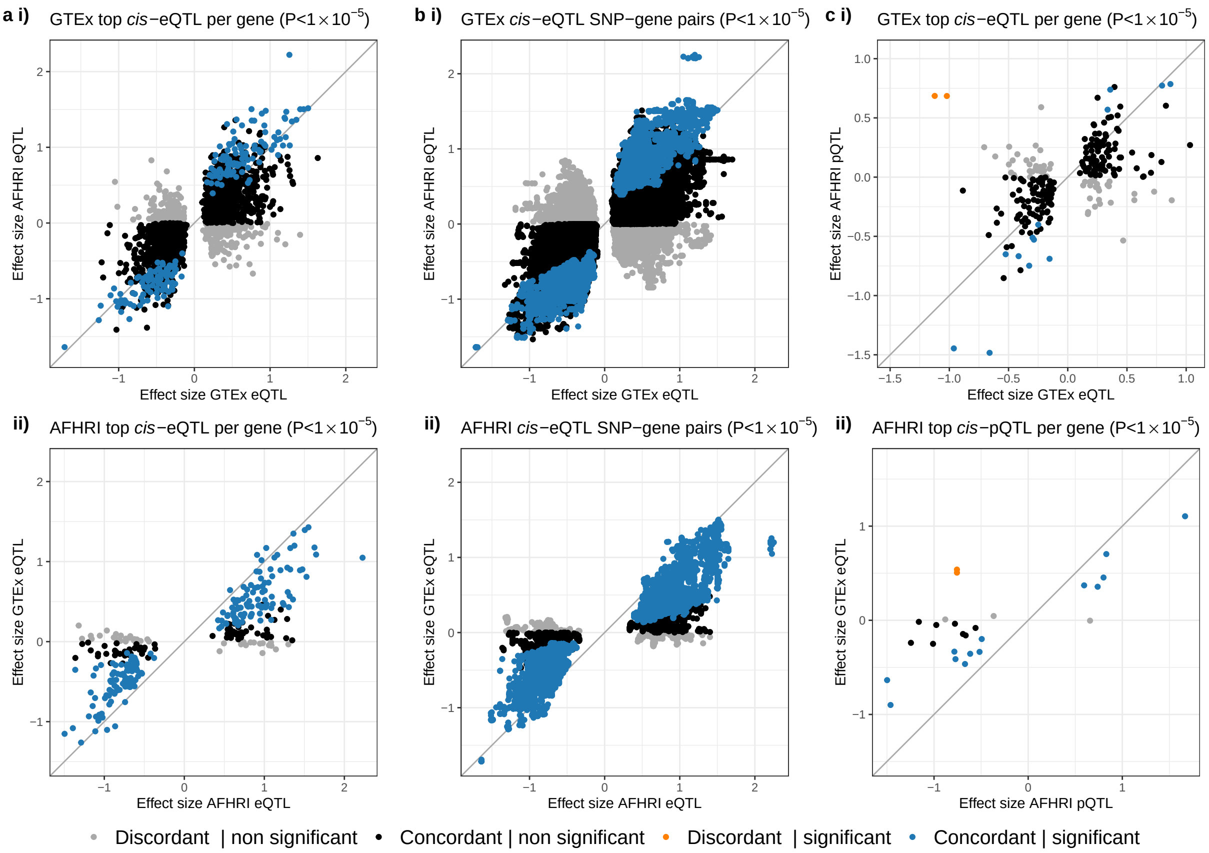

Figure 3: Comparison of *cis*- eQTL and pQTL results to GTEx *cis*-eQTLs in atrial appendage tissue.
a: Comparison of effect sizes of eQTLs in the GTEx and AFHRI cohort for i) the top significant *cis*-eQTL per gene in GTEx $(P < 1 \times 10^{-5})$ and ii) the top significant *cis*-eQTL per gene in AFHRI $(P < 1 \times 10^{-5})$.
b: Comparison of effect sizes of eQTLs in the GTEx and AFHRI cohort for i) all significant *cis*-eQTL SNP-gene pairs in GTEx $(P < 1 \times 10^{-5})$ and ii) all significant *cis*-eQTL SNP-gene pairs in AFHRI $(P < 1 \times 10^{-5})$.
c: Comparison of effect sizes of eQTLs in GTEx and pQTLs in the AFHRI cohort i) the top significant *cis*-eQTL per gene in GTEx $(P < 1 \times 10^{-5})$ and ii) the top significant *cis*-pQTL per gene in AFHRI $(P < 1 \times 10^{-5})$.
All cutoffs refer to uncorrected, nominal P values derived by two-sided tests. Permutation tests were performed for all reported GTEx results, all data from the AFHRI cohort was evaluated by T tests.
eQTL, expression quantitative trait loci; pQTL, protein quantitative trait loci; GTEx, Genotype-Tissue Expression project; Source data are provided as a Source Data file.

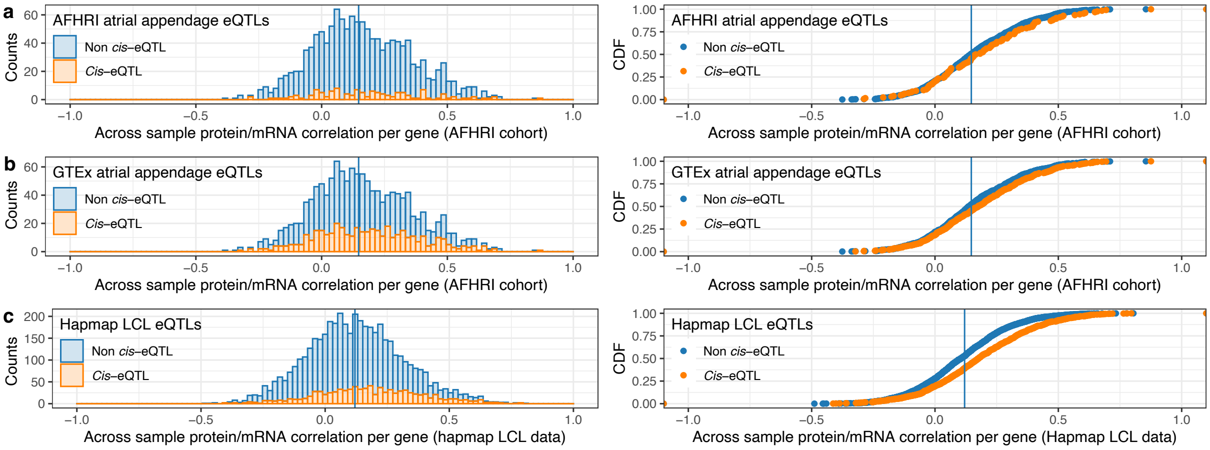

Figure 4: Pearson correlation between transcript and protein levels dependent on *cis*-eQTL annotations in different datasets.

a: AFHRI cohort and *cis*-eQTLs. Histogram and cumulative density function.

b: AFHRI cohort and GTEx *cis*-eQTL annotations. Histogram and cumulative density function.

c: Hapmap LCL data and Hapmap LCL *cis*-eQTL annotations. Histogram and cumulative density function.

eQTL, expression quantitative trait loci; LCL, lymphoblastoid cell line; Source data are provided as a Source Data file.

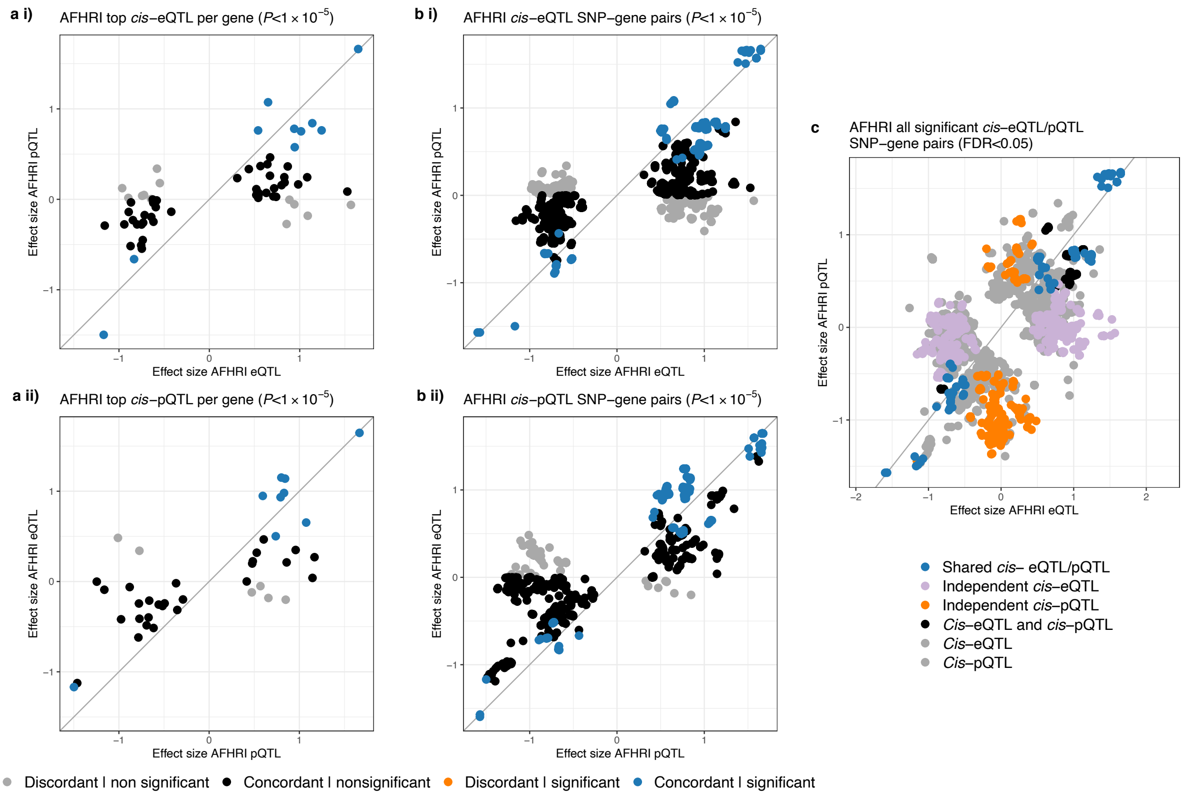

Figure 5: Between-omic comparison of *cis*-quantitative trait loci results.
a: Comparison of effect sizes of eQTLs and pQTLs in the AFHRI cohort for i) the top significant *cis*-eQTL per gene ($P < 1 \times 10^{-5}$) and ii) the top significant *cis*-pQTL per gene ($P < 1 \times 10^{-5}$).
b: Comparison of effect sizes of eQTLs and pQTLs in the AFHRI cohort for i) all significant *cis*-eQTL SNP-gene pairs ($P < 1 \times 10^{-5}$) and ii) all significant *cis*-pQTL SNP-gene pairs ($P < 1 \times 10^{-5}$).
c: Comparison of effect sizes of eQTLs and pQTLs in the AFHRI cohort for all significant *cis*-eQTL and *cis*-pQTL SNP-gene pairs (FDR<0.05, Benjamini-Hochberg procedure).
Nominal P values were derived based on two-sided T tests. FDR correction per omic based on the Benjamini-Hochberg procedure was applied to account for multiple comparisons.
eQTL, expression quantitative trait loci; pQTL, protein quantitative trait loci; SNP, single-nucleotide polymorphism; FDR, false discovery rate; Source data are provided as a Source Data file.

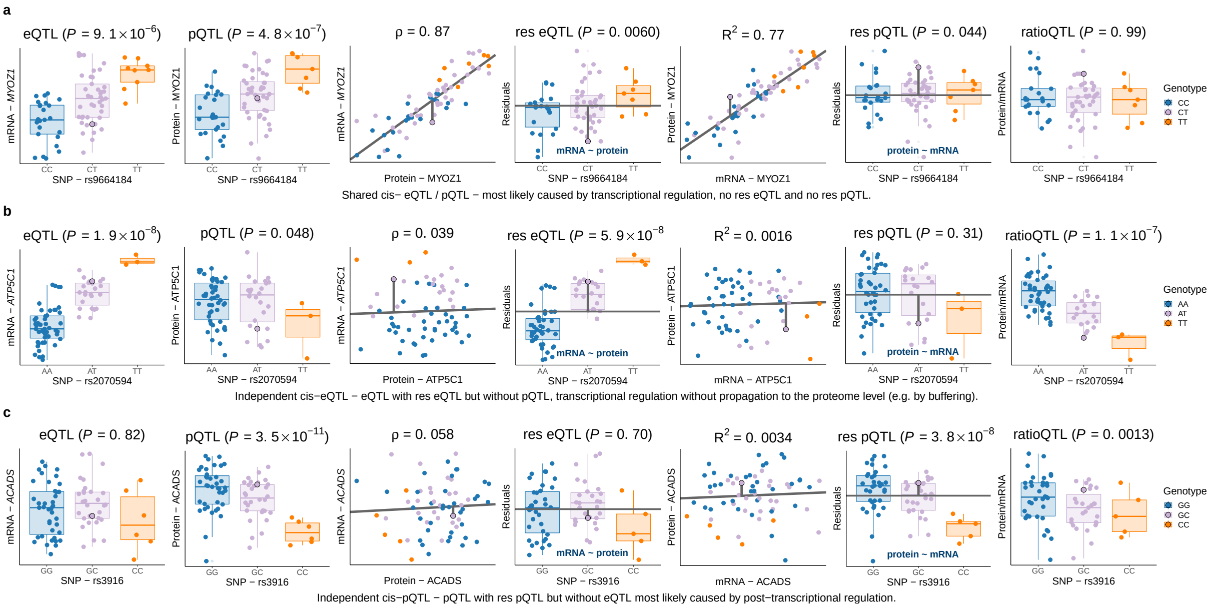

Figure 6: Extended figure for functional *cis*-QTL categories.
Cis-QTL boxplots and scatter plots representing residual derivation and mRNA/protein correlation to visualize the three functional QTL categories. Nominal P values were derived based on two-sided T tests for $N = 75$ (eQTL), $N = 75$ (pQTL) and $N = 66$ (ratioQTL, res eQTL, res pQTL) biologically independent samples. To assess significance, FDR correction per omic based on the Benjamini-Hochberg procedure was applied to account for multiple comparisons.
a: Shared eQTLs/pQTLs represent QTLs, where the effect of transcriptional regulation translates into mRNA and protein abundance exemplified by the significant SNP-gene pair rs9664184-MYOZ1. No corresponding ratioQTL can be observed as the genetic variation is shared across both omics levels.
b: Independent eQTLs depict variants with regulation on mRNA but not on protein level displayed by the significant SNP-transcript pair rs2070594-*ATP5C1*.
c: Independent pQTLs represent variants that show regulation only on protein level as shown for the SNP-protein pair rs3916-ACADS. Genetic influence is not observable on transcript level.
In the boxplots, the lower and upper hinges correspond to the first and third quartiles (the 25th and 75th percentiles). The median is denoted by the central line in the box. The upper/lower whisker extends from the hinge to the largest/smallest value no further than 1.5·IQR from the hinge.
eQTL, expression quantitative trait loci; pQTL, protein quantitative trait loci; res eQTL, expression residual quantitative trait loci; res pQTL, protein residual quantitative trait loci; ratioQTL, ratio quantitative trait loci; IQR, interquartile range;

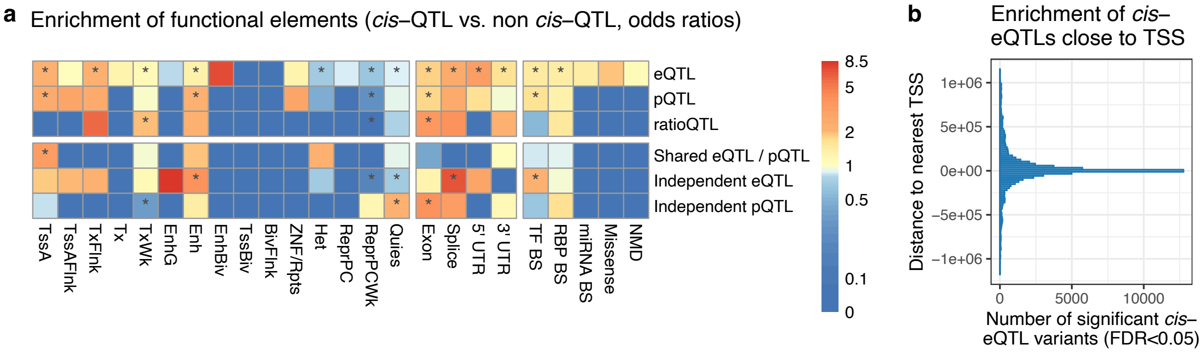

Figure 7: Enrichment of functional elements for different *cis*-QTL categories.
a: Annotations of the top 5 *cis*-QTL hits per gene were compared to a background distribution (100 background SNPs per QTL SNP) matched for MAF and distance to TSS. Displayed are odds ratios that represent enrichment or depletion, stars mark P values < 0.05 for the corresponding Fisher's exact test (two-sided).
b: Enrichment of *cis*-eQTL hits close to the nearest TSS.
QTL, quantitative trait loci; eQTL, expression quantitative trait loci; pQTL, protein quantitative trait loci; ratioQTL, ratio quantitative trait loci; UTR, untranslated region; TF, transcription factor; BS, binding site; NMD, nonsense-mediated decay; TSS, transcription start site; FDR, false discovery rate; Source data are provided as a Source Data file.

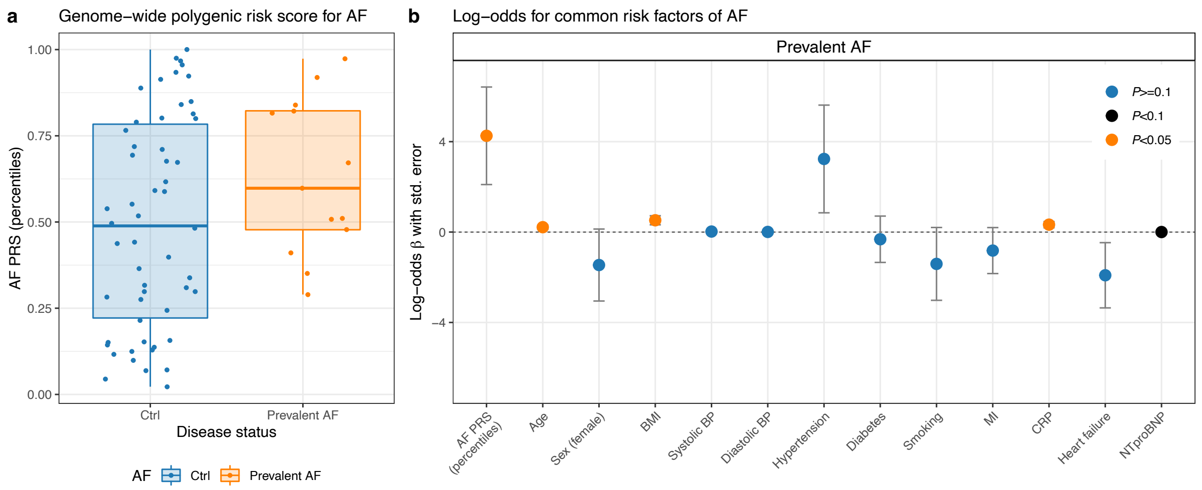

Figure 8: Genome-wide polygenic score adds relevant information in classifying atrial fibrillation disease status.

a: Percentiles of the atrial fibrillation polygenic risk score for controls and prevalent AF cases ($T = -1.8$, $P = 0.043$, one-sided T test, $N = 67$).

b: Logistic regression results for common risk factors of AF. Significant and comparably strong effect for the PRS variable ($\beta = 4.3$, $P = 0.048$, two-sided Z test, $N = 64$, df $= 51$). Data are presented as log-odds ratio +/- standard error.

In the boxplots, the lower and upper hinges correspond to the first and third quartiles (the 25th and 75th percentiles). The median is denoted by the central line in the box. The upper/lower whisker extends from the hinge to the largest/smallest value no further than 1.5·IQR from the hinge.

AF, atrial fibrillation; PRS, genome-wide polygenic score; BMI, body mass index; BP, blood pressure; MI, myocardial infarction; CRP, C-reactive protein; NT-proBNP, N-terminal prohormone of brain natriuretic peptide; IQR, interquartile range; Source data are provided as a Source Data file.

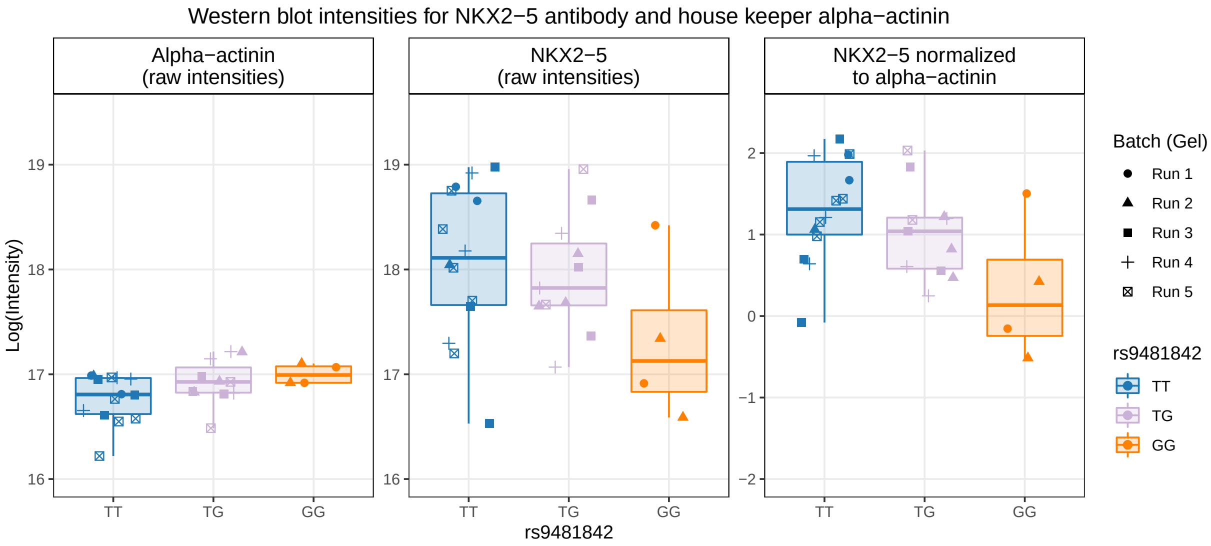

Figure 9: Replication of the NKX2-5 *trans*-eQTL on proteomics level using Western blot analysis.

Association of rs9481842 genotypes with NKX2-5 protein expression and the housekeeper alpha-actinin (5 different Western blots, $N = 29$).

In the boxplots, the lower and upper hinges correspond to the first and third quartiles (the 25th and 75th percentiles). The median is denoted by the central line in the box. The upper/lower whisker extends from the hinge to the largest/smallest value no further than 1.5·IQR from the hinge.

eQTL, expression quantitative trail loci; IQR, interquartile range;

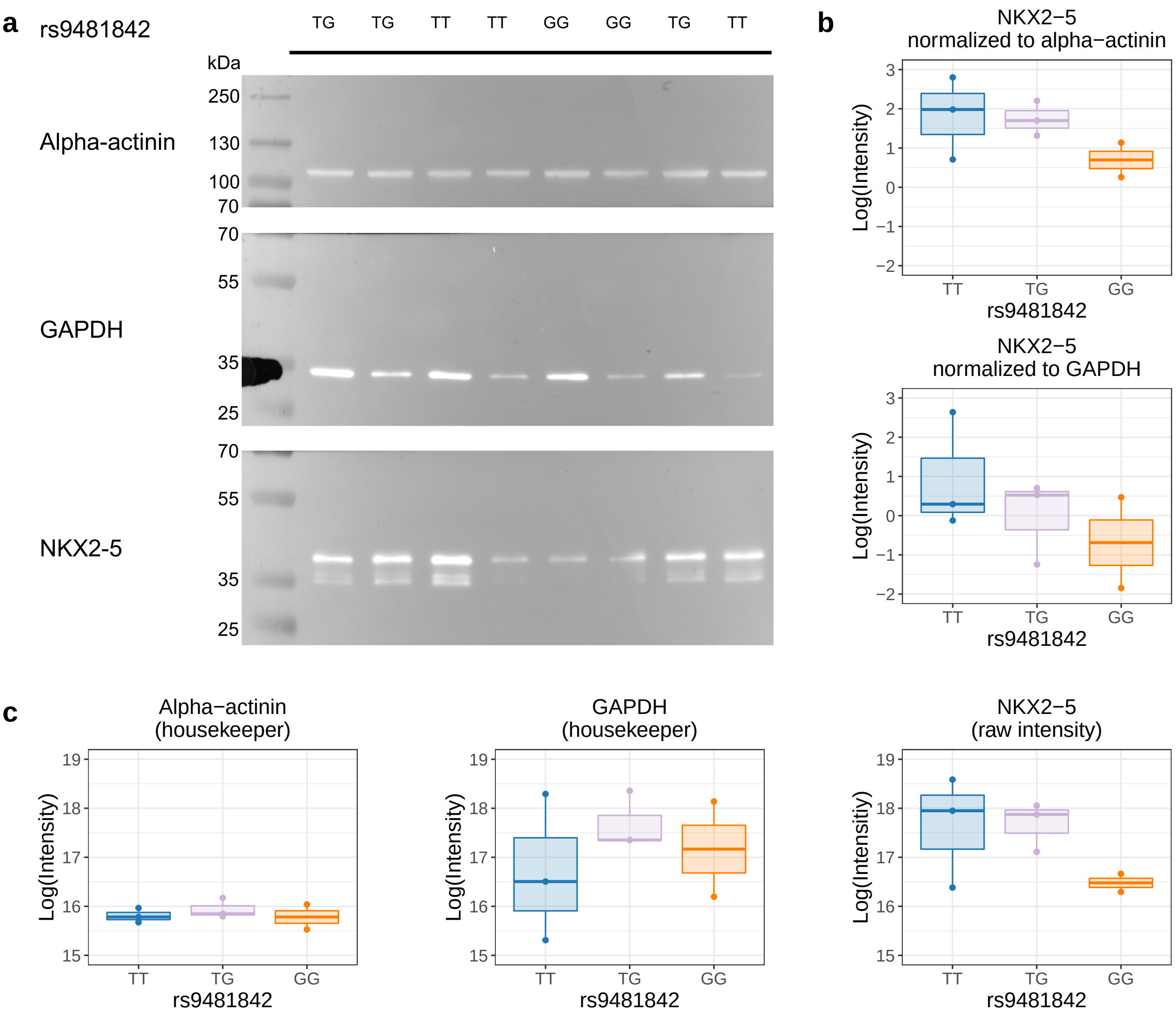

Figure 10: Western blot analysis for NKX2-5 quantification.
a: Exemplary additional Western blot image for alpha-actinin (100 kDa), GAPDH (37 kDa), and NKX2-5 (30-42 kDa) for $N = 8$ biologically independent samples with different genotypes. The membrane was cut in two parts to stain for alpha-actinin and NKX2-5 in parallel. The NKX2-5 membrane was reprobed with GAPDH antibody after incubation with stripping buffer. In total, five Western blots for NKX2-5 and alpha-actinin were performed to measure $N = 29$ biologically independent samples (presented in Fig. 9).
b: Quantification of the NKX2-5 *trans*-protein quantitative trait loci for housekeepers alpha-actinin and GAPDH ($N = 8$ independent biological samples).
c: Raw, absolute quantifications of alpha-actinin, GAPDH and NKX2-5 ($N = 8$ independent biological samples).

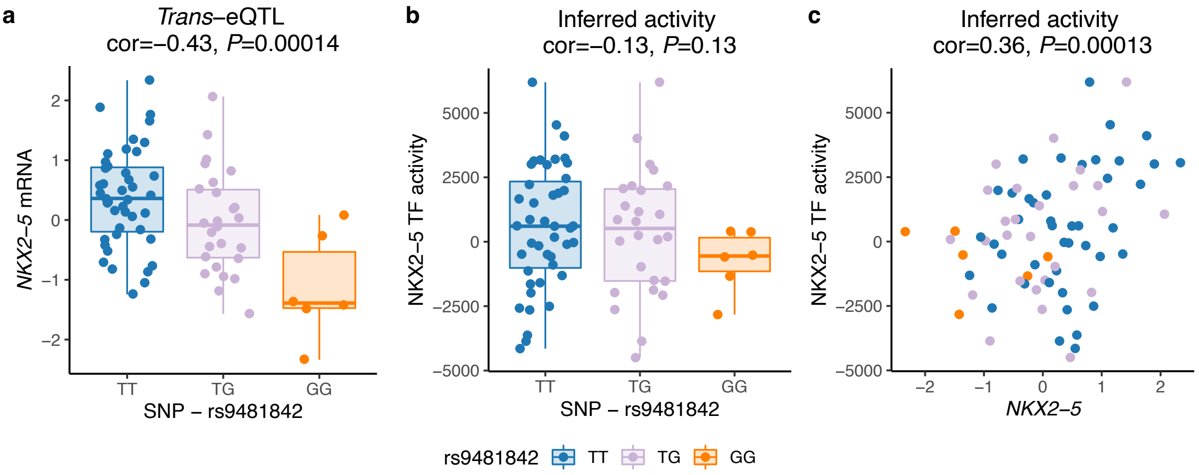

Figure 11: Causal modeling of NKX2-5 and TF activity.
a: *Trans*-eQTL rs948182-*NKX2-5* (two-sided Pearson's correlation).
b: Dependence of TF activity on rs9481842 (one-sided Pearson's correlation).
c: Correlation between *NKX2-5* transcript and TF activity (one-sided Pearson's correlation).
In the boxplots, the lower and upper hinges correspond to the first and third quartiles (the 25th and 75th percentiles). The median is denoted by the central line in the box. The upper/lower whisker extends from the hinge to the largest/smallest value no further than 1.5·IQR from the hinge. $N = 75$ independent biological samples are displayed.
eQTL, expression quantitative trait loci; TF, transcription factor; IQR, interquartile range;

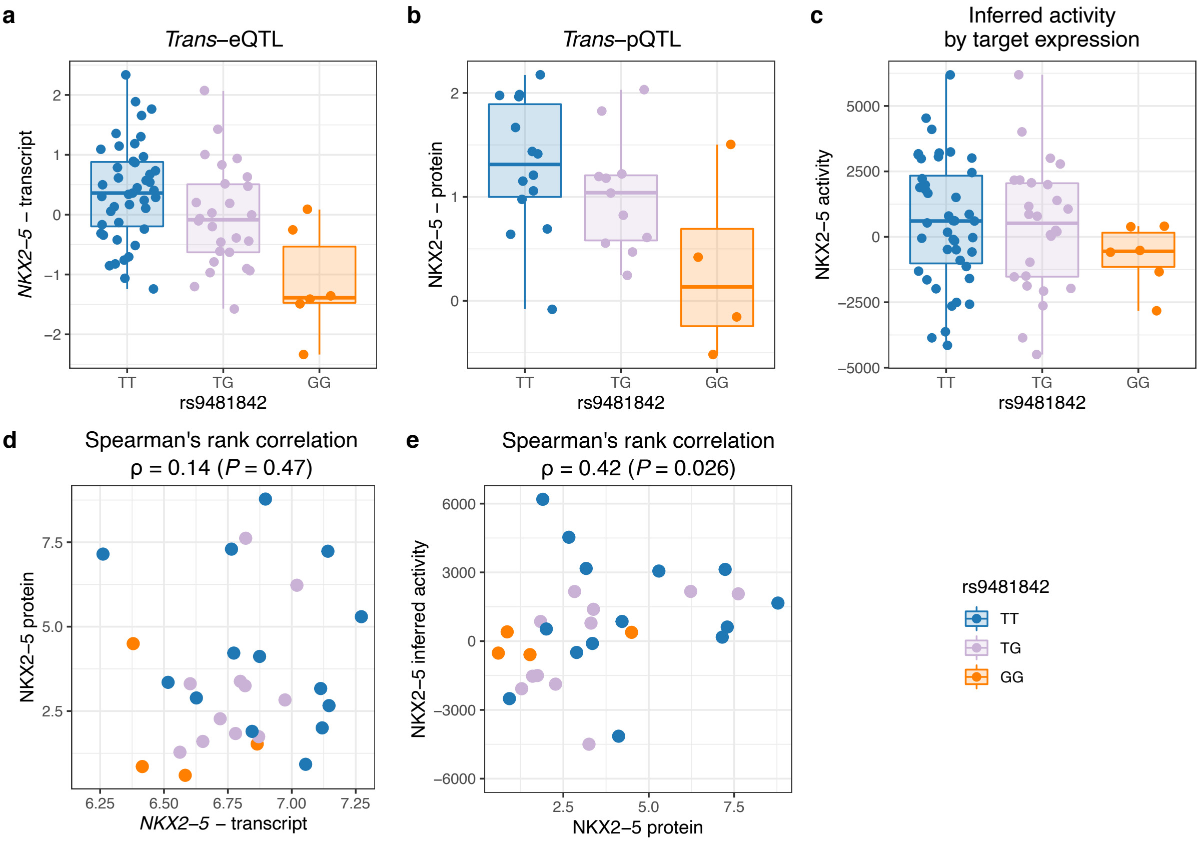

Figure 12: Inferred transcription factory activity strongly correlates with protein intensity.
a: *Trans*-eQTL of the SNP rs9481842 and the transcription factor NKX2-5 discovered by our polygenic risk score based enrichment approach for *trans*-QTL gene candidate selection using microarray mRNA quantifications in $N = 75$ biologically independent samples (two-sided Spearman's rank correlation, $\rho = -0.38$, $P = 0.00086$).
b: *Trans*-pQTL validation of the rs9481842-NKX2-5 *trans*-eQTL in remaining tissue samples using Western blot analysis in $N = 29$ biologically independent samples (two-sided Spearman's rank correlation, $\rho = -0.41$, $P = 0.029$).
c: Estimated transcription factor activity inferred by genome-wide transcriptomics data and independent tissue or cell type specific annotations in $N = 75$ biologically independent samples (one-sided Spearman's rank correlation, $\rho = -0.13$, $P = 0.14$).
d: Correlation between the NKX2-5 mRNA and protein in $N = 29$ biologically independent samples (two-sided Spearman rank correlation).
e: High two-sided Spearman's rank correlation between the inferred NKX2-5 transcription factor activity and actual protein intensities in $N = 29$ biologically independent samples.
In the boxplots, the lower and upper hinges correspond to the first and third quartiles (the 25th and 75th percentiles). The median is denoted by the central line in the box. The upper/lower whisker extends from the hinge to the largest/smallest value no further than 1.5·IQR from the hinge.
eQTL, expression quantitative trait loci; SNP, single-nucleotide polymorphism; QTL, quantitative trait loci; pQTL, protein quantitative trait loci; IQR, interquartile range;

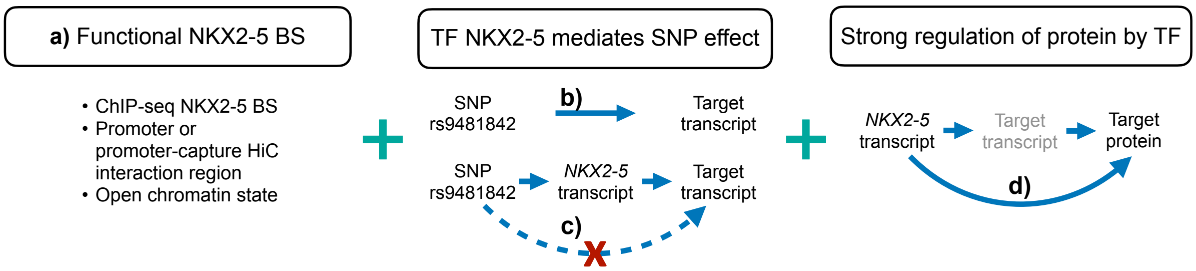

Figure 13: Definition of functional NKX2-5 targets.
Functional NKX2-5 targets were derived in a three step process: first, by the presence of a most likely functional TF BS (a), second by a likely regulation of target transcript by the SNP through the TF (b+c), and finally, we checked for strong regulation of the target protein by the TF (d). TF, transcription factor; BS, binding site; SNP, single-nucleotide polymorphism;

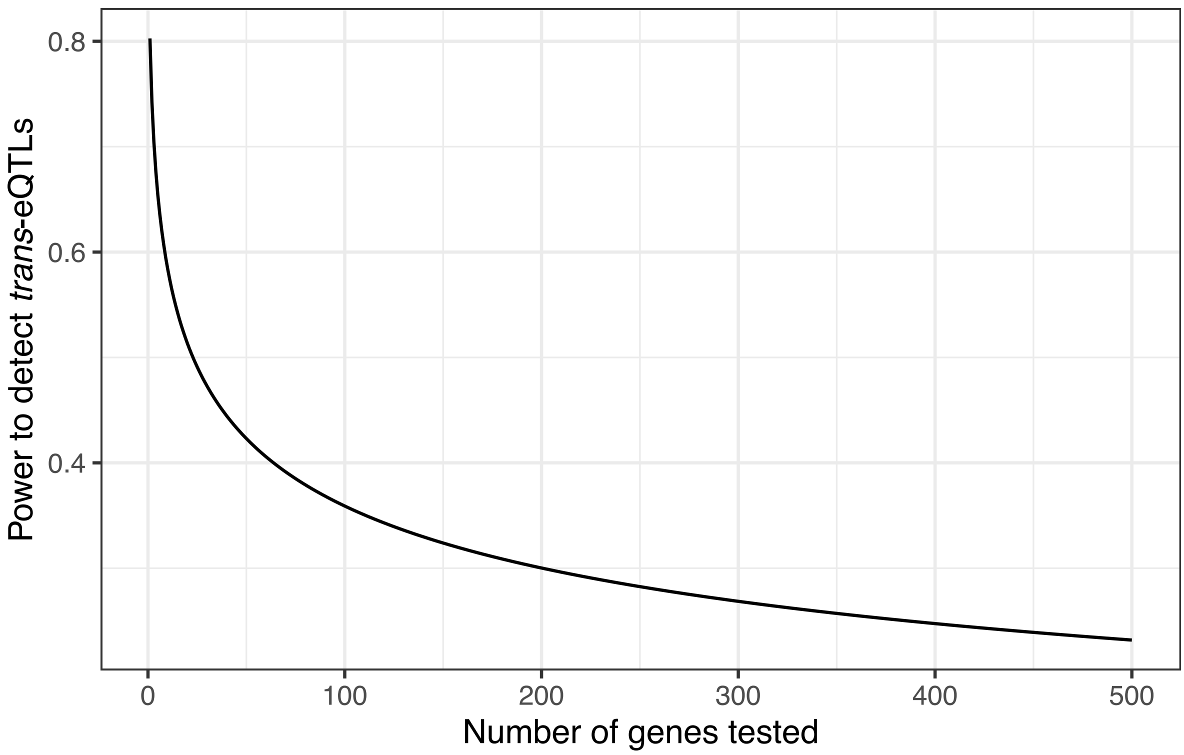

Figure 14: *Trans*-eQTL power analysis.
Power analysis for the strongest *trans*-eQTLs (effect size 21.8%), considering 74 samples, 108 SNPs, $\alpha < 0.05$ and Bonferroni correction. 23 genes correspond to 50% power.
eQTL, expression quantitative trait loci; SNP, single-nucleotide polymorphism; Source data are provided as a Source Data file.

Supplementary Tables

Table 1: Correlation between mRNA and protein for *cis*-eQTL genes.
Comparison of median R^2 and correlation dependent on existing eQTL annotations. Compared are AFHRI genes non *cis*-eQTL vs. *cis*-eQTL genes, AFHRI genes without an *cis*-eQTL in the GTEx atrial appendage data vs. genes with eQTL in GTEx[1] and Hapmap LCL[2] data without and with an eQTL for the LCL computations.
eQTL, expression quantitative trait score; LCL, lymphoblastoid cell line; GTEx, Genotype-Tissue Expression project; Source data are provided as a Source Data file.

	AFHRI cohort		AFHRI cohort		Hapmap LCL data	
**Measure**	**non *cis*-eQTL**	***cis*-eQTL**	**non GTEx *cis*-eQTL**	**GTEx *cis*-eQTL**	**LCL non *cis*-eQTL**	**LCL *cis*-eQTL**
Median(R^2)	0.0265	0.0378	0.0243	0.0354	0.0205	0.0376
Mean(R^2)	0.0634	0.0868	0.0604	0.0771	0.0466	0.0798
Median(correlation)	0.145	0.174	0.137	0.175	0.105	0.176
Mean(correlation)	0.163	0.195	0.156	0.188	0.110	0.185

Table 2: Overlap of *cis*- eQTLs and pQTLs in other studies.
Comparison of *cis*- eQTL/pQTL overlap in previously published studies compared to our dataset. Information about plasma pQTL overlap with GTEx eQTLs is either based on the Sun et al. manuscript (marked with "paper"), or derived from the Sun et al. Supplementary Table S8[3] (marked "suppl.").
eQTL, expression quantitative trait loci; pQTL, protein quantitative trait loci; FDR, false discovery rate; LCL, lymphoblastoid cell line; GTEx, Genotype-Tissue Expression project;

	Overlap of	
**Cohort / datasets**	***cis*-pQTLs with *cis*-eQTLs**	***cis*-eQTLs with *cis*-pQTLs**
AFHRI cohort		
QTL genes	n=21 (24%)	n=21 (17%)
AFHRI cohort		
SNP-gene pairs	n=642 (16%)	n= 642 (14%)
LCL Hause et al.[4]:	none at FDR<0.05	none at FDR<0.05
LCL Battle et al.[2]:		
SNP-gene pairs	67%	35%
Plasma pQTL Sun et al.[3] to all GTEx tissues:		
Lead SNPs for same gene, high LD regions ($r^2 \geq 0.8$)	n=224 (40%) (paper)	
All sentinel SNPs listed in the Sun et al. supplements	n=320 (∼40%) (suppl.)	
Plasma pQTL Sun et al.[3] to GTEx whole blood:		*cis*-eQTL: $P < 1.5\times10^{-11}$
Lead SNPs for same gene, high LD regions ($r^2 \geq 0.8$)	n=117 (19%) (paper)	12.2% (paper)
All sentinel SNPs listed in the Sun et al. supplements	n=152 (∼19%) (suppl.)	
Plasma pQTL Sun et al.[3] to GTEx heart tissue:		
Lead SNPs for same gene, high LD regions ($r^2 \geq 0.8$)		
All sentinel SNPs listed in the Sun et al. supplements	n=60 (∼7.5%) (suppl.)	
Plasma pQTLSun et al.[3] to GTEx liver:		*cis*-eQTL: $P < 1.5\times10^{-11}$
Lead SNPs for same gene, high LD regions ($r^2 \geq 0.8$)	n=70 () (paper)	14.8% (paper)
All sentinel SNPs listed in the Sun et al. supplements	n=126 (∼16%) (suppl.)	
Plasma pQTL Sun et al.[3] to GTEx monocytes:		*cis*-eQTL: $P < 1.5\times10^{-11}$
Lead SNPs for same gene, high LD regions ($r^2 \geq 0.8$)	n=52 () (paper)	14.7% (paper)
All sentinel SNPs listed in the Sun et al. supplements	n=94 (∼12%) (suppl.)	

Table 3: Summary of tested data and discovered *cis*-quantitative trait loci.
Significant *cis*-QTLs in heart atrial appendage tissue for mRNA and protein measurements. Two-sided T tests were evaluated and FDR (according to Benjamini-Hochberg procedure) was assessed per omic to account for multiple comparisons. Results for FDR<0.05 and $P < 1 \times 10^{-5}$ are listed. Loci denote the number of independent loci derived by LD-clumping.
QTL, quantitative trait loci; FDR, false discovery rate; LD, linkage disequilibrium; eQTL, expression quantitative trait loci; pQTL, protein quantitative trait loci; ratioQTL, ratio quantitative trait loci; res eQTL, residual expression quantitative trait loci; res pQTL, residual protein quantitative trait loci; N, sample size; Source data are provided as a Source Data file.

	Tested:			FDR<0.05:			$P<1\times10^{-5}$:			
	**SNPs**	**Pairs**	**Genes**	**Pairs**	**Genes**	**Loci**	**Pairs**	**Genes**	**Loci**	**N**
eQTL	4,861,118	56,139,851	16,306	57,403	1,058	1,657	40,267	552	870	75
pQTL	2,323,504	4,508,654	1,337	4,081	91	139	2,543	45	71	75
ratioQTL	2,249,758	4,198,168	1,243	563	16	23	575	18	27	66
res eQTL	2,249,758	4,198,168	1,243	2,261	63	99	1,504	41	62	66
res pQTL	2,249,758	4,198,168	1,243	1,316	34	45	1,194	29	38	66
Shared eQTL/pQTL	2,249,758	4,198,168	1,243	430	11	14				66
Independent eQTL	2,249,758	4,198,168	1,243	1,593	37	62				66
Independent pQTL	2,249,758	4,198,168	1,243	1,083	21	25				66

Table 4: *Cis*-QTLs overlapping with GWAS hits.
Number of significant *cis*-QTLs (FDR < 0.05) overlapping with variants annotated to cardiovascular traits in the GWAS catalog (or proxy with R^2>0.8).
QTL, quantitative trait loci; GWAS, genome-wide association study; COPD, Chronic obstructive pulmonary disease; eQTL, expression quantitative trait loci; pQTL, protein quantitative trait loci; ratioQTL, ratio quantitative trait loci; res pQTL, protein residual quantitative trait loci; res eQTL, expression residual quantitative trait loci; Source data are provided as a Source Data file.

Trait	eQTL	pQTL	ratioQTL	res pQTL	res eQTL
Atrial fibrillation	15	3	0	0	0
Pulse pressure	12	1	0	0	0
Coronary artery disease	5	0	0	0	0
QT interval	5	0	0	0	0
Creatine kinase levels	1	1	1	1	0
COPD or resting heart rate (pleiotropy)	2	1	0	0	0
PR interval	3	0	0	0	0
Serum uric acid levels	2	0	0	0	1
Incident atrial fibrillation	1	1	0	0	0
Large artery stroke	2	0	0	0	0
Sudden cardiac arrest	2	0	0	0	0
Age-related disease endophenotypes	1	0	0	0	0
Birdshot chorioretinopathy	1	0	0	0	0
Carotid plaque burden (smoking interaction)	1	0	0	0	0
Circulating myeloperoxidase levels (serum)	1	0	0	0	0
Conotruncal heart defects (inherited effects)	1	0	0	0	0
Coronary artery disease (...)	1	0	0	0	0
Heart rate	1	0	0	0	0
Hematology traits	1	0	0	0	0
Homocysteine levels	1	0	0	0	0
Ischemic stroke	0	0	0	1	0
Left atrial antero-posterior diameter	1	0	0	0	0
Migraine	1	0	0	0	0
Peripheral arterial disease (...)	1	0	0	0	0
Prevalent atrial fibrillation	1	0	0	0	0
QRS complex (Sokolow-Lyon)	1	0	0	0	0
Resting heart rate	1	0	0	0	0
RR interval (heart rate)	1	0	0	0	0
Venous thromboembolism	1	0	0	0	0

Table 5: Overlap of *cis*-QTLs with GWAS loci for atrial fibrillation.
QTL hits that overlap with GWAS hits for atrial fibrillation in the GWAS catalog (or proxy with R^2<0.8).
QTL, quantitative trait loci; GWAS, genome-wide association study; eQTL, expression quantitative trait loci; pQTL, protein quantitative trait loci; Source data are provided as a Source Data file.

Region	QTL variant	Reported variant	Reported gene	QTL gene	QTL type	Pubmed-ID	First author
2p13.3	rs13028508	rs10165883	*SNRNP27*	*SNRNP27*	eQTL	29892015	Roselli C
10q22.2	rs10824026	rs10824026	*SYNPO2L*	*MYOZ1*	eQTL	29290336	Nielsen JB
3q22.3	rs6791611	rs1278493	*PPP2R3A*	*PCCB*	eQTL	30061737	Nielsen JB
5q31.2	rs9327807	rs2040862	*WNT8A, NPY6R,* M*YOT, FAM13B*	*FAM13B*	eQTL	30061737	Nielsen JB
2q33.1	rs159321	rs295114	*SPATS2L*	*SPATS2L*	eQTL	29892015	Roselli C
2q33.1	rs1347551	rs3820888	*SPATS2L*	*SPATS2L*	eQTL	30061737	Nielsen JB
3q26.33	rs2339798	rs4855074	*GNB4*	*GNB4*	eQTL	29892015	Roselli C
3q26.33	rs2339798	rs4855075	*GNB4*	*GNB4*	eQTL	29892015	Roselli C
1q32.1	rs951366	rs4951258	*NUCKS1, SLC41A1*	*NUCKS1*	eQTL	30061737	Nielsen JB
1q32.1	rs951366	rs4951261	*NUCKS1*	*NUCKS1*	eQTL	29892015	Roselli C
10q22.2	rs3740293	rs60212594	*SYNPO2L*	*MYOZ1*	eQTL	29892015	Roselli C
10q22.2	rs10824026	rs6480708	*SYNPO2L*	*MYOZ1*	eQTL	29892015	Roselli C
2p13.3	rs13028508	rs6546550	*ANXA4*/*GMCL1*	*SNRNP27*	eQTL	28416818	Christophersen IE
2p13.3	rs13028508	rs6546553	*GMCL1*	*SNRNP27*	eQTL	29892015	Roselli C
2p13.3	rs13028508	rs6747542	*GMCL1, ANXA4*	*SNRNP27*	eQTL	30061737	Nielsen JB
10q22.2	rs10824026	rs7394190	*SYNPO2L*	*MYOZ1*	eQTL	28416818	Christophersen IE
1q32.1	rs951366	rs951366	*NUCKS1*	*NUCKS1*	eQTL	29892015	Roselli C
10q22.2	rs10824026	rs10824026	*SYNPO2L*	*MYOZ1*	pQTL	28416818	Christophersen IE
10q22.2	rs3740293	rs60212594	*SYNPO2L*	*MYOZ1*	pQTL	29892015	Roselli C
10q22.2	rs12570126	rs6480708	*SYNPO2L*	*MYOZ1*	pQTL	29892015	Roselli C
10q22.2	rs12570126	rs7394190	*SYNPO2L*	*MYOZ1*	pQTL	28416818	Christophersen IE

Table 6: Top eQTS genes.
Linear regression results of transcript expression associating with the polygenic risk score for AF. Two-sided T tests were derived from a linear model with covariates and *cis*-SNPs. As T values were further used for ranking genes and not to assess statistical significance, no adjustments for multiple comparisons were applied.
eQTS, expression quantitative trait score; AF, atrial fibrillation; SNP, single-nucleotide polymorphism; N, sample size; df, degrees of freedom; Source data are provided as a Source Data file.

Gene	β	*T* value	*P* value	N	df
LOC105377927	0.311	4.10	0.000116	74	65
SEMA3F	-0.254	-3.95	0.000196	74	65
LOC105371436	-0.306	-3.89	0.000240	74	65
STEAP3	-0.343	-3.80	0.000326	74	65
FBXL7	0.201	3.69	0.000465	74	65
VTN	-0.415	-3.68	0.000476	74	64
WDR82	0.159	3.66	0.000501	74	65
MIR544A	0.577	3.62	0.000570	74	65
NR1I2	0.266	3.59	0.000639	74	65
SNORD91A	0.384	3.58	0.000664	74	65
PLCL1	0.383	3.57	0.000668	74	65
LOC105374629	0.218	3.53	0.000758	74	65
LOC729080	0.282	3.53	0.000780	74	65
OIP5-AS1	0.225	3.52	0.000805	74	65
TXNDC12-AS1	-0.330	-3.50	0.000846	74	65
CRELD2	-0.189	-3.48	0.000896	74	65
CEP85L	0.226	3.45	0.00100	74	65
FKBP11	-0.330	-3.41	0.00112	74	65
SLC22A24	-0.268	-3.39	0.00119	74	65
VAT1L	-0.520	-3.39	0.00119	74	65
TFIP11	0.242	3.37	0.00128	74	65
LOC105377151	0.283	3.36	0.00131	74	65
TRIM43	0.239	3.36	0.00132	74	65
MIR376A2	0.481	3.35	0.00133	74	65
UPK1A-AS1	0.277	3.35	0.00134	74	65
WAC-AS1	0.226	3.33	0.00142	74	65
LOC101928257	0.212	3.33	0.00145	74	65
LOC105374674	0.230	3.31	0.00152	74	65
LINCR-0002	-0.196	-3.31	0.00152	74	65
FAM78A	0.219	3.31	0.00152	74	64
CFAP57	-0.232	-3.30	0.00157	74	65
TMPRSS9	-0.221	-3.29	0.00163	74	65
KLKB1	0.616	3.27	0.00171	74	65
RUNDC3A-AS1	0.255	3.27	0.00173	74	65
SGK2	-0.275	-3.24	0.00191	74	65
C1orf56	0.273	3.23	0.00195	74	65

Table 7: Enriched GO terms for eQTS GSEA.
GSEA results on eQTS T value rankings. Shown are all GO terms enriched at FDR<0.05 (Benjamini-Hochberg procedure to account for multiple comparisons of the two-sided, permutation-derived P values). Size refers to the number of genes in the gene set after removing those not evaluated for eQTS.Full summary statistics on all tables are available under `https://doi.org/10.5281/zenodo.5080229`
GO, Gene Ontology; eQTS, expression quantitative trait score; GSEA, gene set enrichment analysis; NES, normalized enrichment score; FDR, false discovery rate; Source data are provided as a Source Data file.

GO ID	GO term	NES	*P* value	FDR	Size
GO:0006091	Generation of precursor metabolites and energy	2.03	1.61×10^{-5}	0.00397	268
GO:0003012	Muscle system process	1.85	1.61×10^{-5}	0.00397	264
GO:0015980	Energy derivation by oxidation of organic compounds	2.21	1.65×10^{-5}	0.00397	197
GO:0045333	Cellular respiration	2.4	1.69×10^{-5}	0.00397	129
GO:0022900	Electron transport chain	2.17	1.74×10^{-5}	0.00397	86
GO:0003015	Heart process	2.17	1.75×10^{-5}	0.00397	80
GO:0006119	Oxidative phosphorylation	2.35	1.76×10^{-5}	0.00397	75
GO:0006942	Regulation of striated muscle contraction	2.13	1.76×10^{-5}	0.00397	74
GO:0055117	Regulation of cardiac muscle contraction	2.11	1.77×10^{-5}	0.00397	63
GO:0033108	Mitochondrial respiratory chain complex assembly	2.26	1.78×10^{-5}	0.00397	57
GO:0097031	Mitochondrial respiratory chain complex I biogenesis	2.38	1.80×10^{-5}	0.00397	46
GO:0009060	Aerobic respiration	2.32	1.80×10^{-5}	0.00397	48
GO:0002455	Humoral immune response mediated by circulating immunoglobulin	-2.22	2.25×10^{-5}	0.00397	46
GO:0006956	Complement activation	-2.27	2.27×10^{-5}	0.00397	52
GO:0019724	B cell mediated immunity	-2.02	2.32×10^{-5}	0.00397	76
GO:0072599	Establishment of protein localization to endoplasmic reticulum	-2.29	2.36×10^{-5}	0.00397	90
GO:0030216	Keratinocyte differentiation	-1.99	2.37×10^{-5}	0.00397	97
GO:0070972	Protein localization to endoplasmic reticulum	-2.07	2.40×10^{-5}	0.00397	109
GO:0006413	translational initiation	-1.93	2.44×10^{-5}	0.00397	127
GO:0006612	Protein targeting to membrane	-2.03	2.46×10^{-5}	0.00397	141
GO:0006959	Humoral immune response	-1.77	2.48×10^{-5}	0.00397	153
GO:0008544	Epidermis development	-1.69	2.60×10^{-5}	0.00398	244
GO:1903034	Regulation of response to wounding	-1.57	2.80×10^{-5}	0.0041	386
GO:0090257	Regulation of muscle system process	1.79	3.31×10^{-5}	0.00465	184
GO:2000257	Regulation of protein activation cascade	-2.16	4.43×10^{-5}	0.00537	33
GO:0031424	Keratinization	-2.05	4.51×10^{-5}	0.00537	48
GO:0002920	Regulation of humoral immune response	-2.07	4.51×10^{-5}	0.00537	47
GO:0072376	Protein activation cascade	-1.95	4.63×10^{-5}	0.00537	74
GO:0072521	Purine-containing compound metabolic process	1.59	4.70×10^{-5}	0.00537	356
GO:0006936	Muscle contraction	1.79	4.91×10^{-5}	0.00537	217
GO:0009141	Nucleoside triphosphate metabolic process	1.79	4.95×10^{-5}	0.00537	201
GO:0072350	Tricarboxylic acid metabolic process	2.11	5.43×10^{-5}	0.00571	37
GO:0006941	Striated muscle contraction	1.93	6.95×10^{-5}	0.00706	90
GO:1903115	Regulation of actin filament-based movement	2.08	7.28×10^{-5}	0.00706	32
GO:0009913	Epidermal cell differentiation	-1.82	7.34×10^{-5}	0.00706	137
GO:0006937	Regulation of muscle contraction	1.84	8.45×10^{-5}	0.0079	139
GO:0048738	Cardiac muscle tissue development	1.82	1.02×10^{-4}	0.00924	129
GO:0055006	Cardiac cell development	1.99	1.08×10^{-4}	0.00955	47
GO:0010881	Regulation of cardiac muscle contraction by regulation of the release of sequestered calcium ion	2.08	1.11×10^{-4}	0.00962	18
GO:0000184	Nuclear-transcribed mRNA catabolic process, nonsense-mediated decay	-1.8	1.19×10^{-4}	0.01	103
GO:0050727	Regulation of inflammatory response	-1.59	1.33×10^{-4}	0.0109	274
GO:0086004	Regulation of cardiac muscle cell contraction	2.01	1.47×10^{-4}	0.0117	27
GO:0002443	Leukocyte mediated immunity	-1.69	1.49×10^{-4}	0.0117	160
GO:0090150	Establishment of protein localization to membrane	-1.6	1.56×10^{-4}	0.0119	241
GO:0000209	Protein polyubiquitination	1.65	1.63×10^{-4}	0.0122	229
GO:0055086	Nucleobase-containing small molecule metabolic process	1.47	1.83×10^{-4}	0.0134	476
GO:0042787	Protein ubiquitination involved in ubiquitin-dependent protein catabolic process	1.75	2.20×10^{-4}	0.0155	126

GO ID	GO term	NES	*P* value	FDR	Size
GO:0010882	Regulation of cardiac muscle contraction by calcium ion signaling	2.03	2.21×10^{-4}	0.0155	22
GO:0098901	Regulation of cardiac muscle cell action potential	2.01	2.41×10^{-4}	0.0165	19
GO:1901657	Glycosyl compound metabolic process	1.56	2.53×10^{-4}	0.017	327
GO:0002673	Regulation of acute inflammatory response	-1.84	2.78×10^{-4}	0.018	69
GO:0009123	Nucleoside monophosphate metabolic process	1.63	2.78×10^{-4}	0.018	219
GO:0030855	Epithelial cell differentiation	-1.43	2.89×10^{-4}	0.0184	469
GO:0008016	Regulation of heart contraction	1.61	3.29×10^{-4}	0.0201	205
GO:0003013	Circulatory system process	1.53	3.31×10^{-4}	0.0201	343
GO:0070252	Actin-mediated cell contraction	1.86	3.35×10^{-4}	0.0201	70
GO:0030048	Actin filament-based movement	1.78	3.65×10^{-4}	0.0213	89
GO:0010880	Regulation of release of sequestered calcium ion into cytosol by sarcoplasmic reticulum	1.97	3.68×10^{-4}	0.0213	24
GO:0002027	Regulation of heart rate	1.82	4.37×10^{-4}	0.0249	82
GO:0060306	Regulation of membrane repolarization	1.94	4.75×10^{-4}	0.0267	28
GO:0040029	Regulation of gene expression, epigenetic	1.61	5.30×10^{-4}	0.0292	193
GO:0043588	Skin development	-1.57	6.11×10^{-4}	0.0332	202
GO:0002064	Epithelial cell development	-1.58	6.54×10^{-4}	0.0349	177
GO:0044033	Multi-organism metabolic process	-1.66	6.80×10^{-4}	0.0358	121
GO:0045165	Cell fate commitment	-1.54	6.95×10^{-4}	0.036	218
GO:0061448	Connective tissue development	-1.57	7.31×10^{-4}	0.0367	183
GO:0060968	Regulation of gene silencing	1.85	7.37×10^{-4}	0.0367	47
GO:0002063	Chondrocyte development	-1.99	7.42×10^{-4}	0.0367	21
GO:0033561	Regulation of water loss via skin	-2	7.80×10^{-4}	0.0381	18
GO:0002066	Columnar/cuboidal epithelial cell development	-1.86	8.11×10^{-4}	0.0385	45
GO:0009896	Positive regulation of catabolic process	1.46	8.12×10^{-4}	0.0385	372
GO:0060537	Muscle tissue development	1.52	8.42×10^{-4}	0.0388	252
GO:0061136	Regulation of proteasomal protein catabolic process	1.6	8.50×10^{-4}	0.0388	167
GO:0018149	Peptide cross-linking	-1.82	8.63×10^{-4}	0.0388	54
GO:0061035	Regulation of cartilage development	-1.82	8.65×10^{-4}	0.0388	57
GO:0099623	Regulation of cardiac muscle cell membrane repolarization cytosol by sarcoplasmic reticulum	1.92	9.64×10^{-4}	0.0427	19
GO:0061061	Muscle structure development	1.44	9.92×10^{-4}	0.0429	399
GO:1903522	Regulation of blood circulation	1.5	9.95×10^{-4}	0.0429	273
GO:0045669	Positive regulation of osteoblast differentiation	-1.8	1.02×10^{-3}	0.0437	56
GO:0050818	Regulation of coagulation	-1.7	1.05×10^{-3}	0.0439	85
GO:0014013	Regulation of gliogenesis	-1.71	1.06×10^{-3}	0.0439	87

Table 8: Top pQTS genes.
Linear regression results of protein expression associating with the polygenic risk score for AF. Two-sided T tests were derived from a linear model with covariates and *cis*-SNPs. As T values were further used for ranking genes and not to assess statistical significance, no adjustment for multiple comparisons was applied.
pQTS, protein quantitative trait score; AF, atrial fibrillation; SNP, single-nucleotide polymorphism; N, sample size; df, degrees of freedom; Source data are provided as a Source Data file.

Gene	β	*T* value	*P* value	*N*	df
NAMPT	-0.158	-3.07	0.00315	73	63
MARK1	-0.176	-2.58	0.0121	73	64
GYG1	0.0941	2.57	0.0125	73	64
AOC3	0.157	2.51	0.0146	73	64
PGM2	-0.184	-2.46	0.0167	73	64
CD36	0.0986	2.45	0.0171	73	64
CDIPT	0.126	2.40	0.0195	73	64
RPL37AP8	0.214	2.35	0.0219	73	64
RPL37L	0.214	2.35	0.0219	73	64
RAB2A	0.113	2.32	0.0235	73	64
RPS4X	0.102	2.27	0.0263	73	64
RPL39	-0.148	-2.23	0.0292	73	64
MYO1C	0.0818	2.20	0.0311	73	64
PCBP2	-0.0961	-2.20	0.0314	73	64
ERP44	-0.0954	-2.17	0.0337	73	64
BLVRA	-0.110	-2.15	0.0350	73	64
NIPSNAP3A	-0.0955	-2.13	0.0376	73	61
XRCC6	-0.0651	-2.06	0.0431	73	64
CLIC4	-0.0771	-2.04	0.0460	73	64
BCL11B	0.139	2.02	0.0479	73	64
TALDO1	-0.0884	-2.01	0.0488	73	64
MAP1LC3B	0.121	2.00	0.0495	73	64
MAP1LC3B2	0.121	2.00	0.0495	73	64
RPL13A	-0.170	-2.00	0.0498	73	64

Table 9: Enriched GO term for pQTS GSEA.
GSEA results on pQTS T value rankings. Shown is the GO term enriched at FDR<0.05 (Benjamini-Hochberg procedure to account for multiple comparisons of the two-sided, permutation-derived P values). Size refers to the number of genes in the gene set after removing those not evaluated for pQTS.
GO, Gene Ontology; pQTS, protein quantitative trait score; GSEA, gene set enrichment analysis; NES, normalized enrichment score; FDR, false discovery rate; Source data are provided as a Source Data file.

GO ID	GO term	NES	*P* value	FDR	Size
GO:0044281	Small molecule metabolic process	-1.68	1.34×10^{-5}	0.0270	332

Table 10: Transcriptomics core gene candidates extracted from eQTS GSEA leading edge. Transcripts that appeared in 14 or more GSEA (on eQTS) leading edges for enriched GO terms (FDR<0.05).
eQTS, expression quantitative trait score; GSEA, gene set enrichment analysis; #, number of times that the gene appeared in the leading edge; Source data are provided as a Source Data file.

Gene	#	Gene	#	Gene	#	Gene	#	Gene	#
ANK2	23	*MYH7*	21	*DMD*	18	*NKX2-5*	15	*NDUFS6*	14
RYR2	23	*CACNA1C*	20	*SCN5A*	18	*TNNT2*	15	*NDUFV2*	14
CAV3	22	*PKP2*	20	*KCNJ2*	17	*CALM1*	14	*TNNI3*	14
ATP1A2	21	*TAZ*	20	*PLN*	17	*CALM3*	14		
GJA5	21	*SLC8A1*	19	*MYL2*	16	*MYBPC3*	14		

Table 11: Proteomics core gene candidates extracted from pQTS GSEA leading edge. Proteins that appeared in the leading edge of the significantly enriched pQTS gene set (FDR<0.05). pQTS, protein quantitative trait score; GSEA, gene set enrichment analysis; Source data are provided as a Source Data file.

Gene	Gene	Gene	Gene	Gene	Gene	Gene
ABHD10	APOC1	COX5A	GBAS	MPST	NME1	PRELP
ACAD10	AQP1	COX6C	GCDH	MSRA	NT5C	PRKAR2B
ACADVL	ATP1B1	CRAT	GLRX	MTAP	OGDH	PTGES2
ACAT1	ATP5A1	CS	GLUD1	MTHFD1	OGN	PTGR2
ACO1	ATP5D	CYB5R3	GOT1	NAMPT	OLA1	QDPR
ACO2	ATP5F1	CYC1	GPD1L	NDUFA10	OXCT1	SDHA
ACSBG2	ATP5J2	CYGB	GPI	NDUFA3	P4HB	SDHB
ADA	BCAT2	DBT	GSS	NDUFA5	PAFAH1B1	SDHD
ADI1	BDH1	DCXR	GSTO1	NDUFA7	PAM	SLC25A11
ADK	BGN	DDAH2	HIBADH	NDUFA9	PCCA	SLC25A12
AHCY	BRP44	DLAT	HPRT1	NDUFB10	PCCB	STOML2
AK4	C3	ECH1	HSD17B10	NDUFB11	PCYOX1	SUCLA2
AKR1A1	CA1	ECHS1	HSD17B4	NDUFB2	PDE5A	SUCLG1
AKR7A2	CA3	ECI2	IDH2	NDUFB3	PDHA1	TALDO1
ALDH1A1	CAT	ENO1	IDH3A	NDUFB8	PDXK	TPI1
ALDH1A2	CBR1	ENO3	IVD	NDUFB9	PEPD	UQCR10
ALDH2	CKM	ERLIN2	LHPP	NDUFC2	PGK1	UQCRC2
ALDH4A1	COQ3	ESD	MCCC1	NDUFS3	PGM1	UQCRFS1
ALDH5A1	COQ5	ETFA	MCCC2	NDUFS4	PGM2	VCAN
ALDH6A1	COQ7	ETFB	MCEE	NDUFS7	PGM5	WARS
ALDH7A1	COQ9	FH	ME2	NDUFS8	PPA1	
APOBEC2	COX4I1	FMOD	ME3	NDUFV1	PRDX4	

Table 12: Disease annotations for putative core genes and functional targets in literature. Findings relating putative core genes and functional targets to cardiovascular phenotypes. Additionally, as stated by Wang and colleagues[5], multiple genes were either identified by their integrative omics approach or mentioned in the OMIM[6] database.
AF, atrial fibrillation; DCM, Dilated cardiomyopathy; HCM, Hypertrophic cardiomyopathy; OMIM, online Mendelian inheritance in men;
*Mutation known to affect cardiovascular phenotypes; **Mutation known to affect arrhythmias; $^{+}$Differential expression or functional impairment for cardiovascular phenotypes; $^{++}$Differential expression or functional impairment for arrhythmias;

Gene	Finding	Clinical phenotypes	First Author
*TNNT2**$^{,++}$	TNNT2 mutations	DCM	Hershberger[7]
	Lower TNNT2 protein expression (human atrial tissue)	AF	Doll[8]
*NKX2-5***	NKX2-5 mutations including loss of function mutation	Arrhythmia, AF	Jhaveri[9], Huang[10]
NDUFA9^{++}	Impaired complex I function (human atrial tissue)	AF, diabetes	Kanaan[11]
NDUFB3^{+}	NDUFB3 deficiency	Cardiomyopathy	El-Hattab[12]
*MYL4***$^{,++}$	Mutation in MYL4	Familial AF	Orr[13]
	Lower MYL4 protein expression (human atrial tissue)	AF	Doll[8]
CKM^{++}	Lower CKM protein expression (human atrial tissue)	AF	Tu[14], Doll[8]
	Lower CKM protein expression (human myocardial tissue)	HCM	Coats[15] (Coats Suppl. Table 3)
PGAM2^{++}	Lower PGAM2 protein expression (human atrial tissue)	AF	Tu[14]
	Lower PGAM2 protein expression (human myocardial tissue)	HCM	Coats[15] (Coats Suppl. Table 3)
*TNNC1**	Mutation in TNNC1	Cardiomyopathy	Parvatiyar[16]
*ETFB***$^{,++}$	Lower ETFB protein expression (human atrial tissue)	AF	Tu[14]
	ETFB mutation	Arrhythmias, HCM, DCM, conduction defects	Florian[17]
ALDOA^{++}	Lower ALDOA protein expression (human atrial tissue)	AF	Tu[14]
	Lower ALDOA protein expression (human myocardial tissue)	HCM	Coats[15] (Coats Suppl. Table 3)
*TCAP**	TCAP gene mutations	HCM, DCM	Hayashi[18]
TOM1L2	Differentially expressed	AF together with neurocognitive decline	Dalal[19]

Table 13: Putative core genes and functional targets differential expression in the GSE128188 dataset.
RNA-seq differential expression results for AF in right and left atrial appendage tissue. Statistics were calculated using edgeR's exact test and are reported from the original authors of the study. FDR_{core} denotes Benjamini-Hochberg false discovery rate applied only on the 20 putative core genes listed below.
Additionally, as stated by Wang and colleagues[5], multiple genes were either identified by their integrative omics approach or mentioned in the OMIM[6] database.
AF, atrial fibrillation; logFC, logarithm of the fold change; QTL, quantitative trait loci; FDR, false discovery rate;
*Mutation known to affect cardiovascular phenotypes; **Mutation known to affect arrhythmias;
$^{+}$Differential expression or functional impairment for cardiovascular phenotypes; $^{++}$Differential expression or functional impairment for arrhythmias;

		mRNA AF association (right atrial appendage)				mRNA AF association (left atrial appendage)			
**Gene**	**Type**	**logFC**	***P* value**	**FDR**	**FDR_{core}**	**logFC**	***P* value**	**FDR**	**FDR_{core}**
TNNT2$^{*,++}$	*Trans*-eQTL	-0.117	0.382	1.00	0.616	-0.191	0.152	0.983	0.522
*NKX2-5***	*Trans*-eQTL	-0.121	0.431	1.00	0.616	-0.104	0.498	1.00	0.712
CYB5R3	*Trans*-pQTL	-0.00112	0.993	1.00	0.993	0.0529	0.672	1.00	0.829
NDUFB3^{+}	*Trans*-pQTL	-0.293	0.0443	0.554	0.272	-0.113	0.439	1.00	0.701
HIBADH	*Trans*-pQTL	-0.236	0.0817	0.686	0.272	-0.147	0.277	1.00	0.522
NDUFA9^{++}	*Trans*-pQTL	-0.138	0.285	0.971	0.571	-0.0964	0.456	1.00	0.701
DLAT	*Trans*-pQTL	-0.0498	0.735	1.00	0.817	-0.0373	0.800	1.00	0.889
PPIF	NKX2-5 target	-0.0746	0.615	1.00	0.724	0.0145	0.922	1.00	0.922
MYL4$^{**,++}$	NKX2-5 target	-0.549	0.000681	0.0546	0.0136	-0.412	0.0107	0.338	0.214
CKM^{++}	NKX2-5 target	-0.208	0.239	0.941	0.571	-0.194	0.271	1.00	0.522
MYL7	NKX2-5 target	-0.352	0.0731	0.658	0.272	-0.271	0.167	1.00	0.522
PGAM2^{++}	NKX2-5 target	-0.521	0.00292	0.129	0.0292	-0.367	0.0359	0.596	0.359
*TNNC1**	NKX2-5 target	-0.0311	0.833	1.00	0.877	0.167	0.259	1.00	0.522
CYC1	NKX2-5 target	-0.237	0.11	0.77	0.315	-0.158	0.287	1.00	0.522
ETFB$^{**,++}$	NKX2-5 target	-0.0999	0.460	1.00	0.616	-0.0513	0.704	1.00	0.829
PRDX5	NKX2-5 target	-0.226	0.074	0.660	0.272	-0.230	0.0688	0.764	0.459
AK1	NKX2-5 target	-0.136	0.357	0.998	0.616	-0.180	0.225	1.00	0.522
ALDOA^{++}	NKX2-5 target	-0.0992	0.492	1.00	0.616	-0.0568	0.694	1.00	0.829
*TCAP**	NKX2-5 target	-0.164	0.277	0.967	0.571	-0.170	0.259	1.00	0.522
TOM1L2	NKX2-5 target	-0.098	0.493	1.00	0.616	0.0205	0.886	1.00	0.922

Table 14: Putative core genes and functional targets AF disease association for the proteomics dataset PXD006675.

Proteomics differential abundance results in human left atrial appendage tissue for AF. Two-sided T tests were calculated and are reported from the original authors of the study.

AF, atrial fibrillation; QTL, quantitative trait loci; FDR, false discovery rate;

*Mutation known to affect cardiovascular phenotypes; **Mutation known to affect arrhythmias; $^{+}$Differential expression or functional impairment for cardiovascular phenotypes; $^{++}$Differential expression or functional impairment for arrhythmias;

		Protein AF association			
**Gene**	**Type**	**Difference**	***P* value**	**Significant**	**FDR**
TNNT2$^{*,++}$	*Trans*-eQTL	-1.300	0.000973	yes	0.0185
NKX2-5**	*Trans*-eQTL				
CYB5R3	*Trans*-pQTL	0.260	0.113		0.195
NDUFB3^{+}	*Trans*-pQTL	-0.195	0.611		0.726
HIBADH	*Trans*-pQTL	-0.197	0.504		0.638
NDUFA9^{++}	*Trans*-pQTL	-0.075	0.727		0.727
DLAT	*Trans*-pQTL	-0.409	0.0284		0.0674
PPIF	NKX2-5 target	-0.124	0.711		0.727
MYL4$^{**,++}$	NKX2-5 target	-1.480	0.00594	yes	0.0376
CKM^{++}	NKX2-5 target	-0.955	0.00499	yes	0.0376
MYL7	NKX2-5 target	-0.771	0.0188		0.0562
PGAM2^{++}	NKX2-5 target	-0.776	0.0207		0.0562
TNNC1*	NKX2-5 target	0.293	0.692		0.727
CYC1	NKX2-5 target	-0.723	0.0187		0.0562
ETFB$^{**,++}$	NKX2-5 target	0.562	0.127		0.201
PRDX5	NKX2-5 target	-0.647	0.0142		0.0562
AK1	NKX2-5 target	-0.609	0.0561		0.107
ALDOA^{++}	NKX2-5 target	-0.644	0.0391		0.0825
TCAP*	NKX2-5 target	0.531	0.360		0.489
TOM1L2	NKX2-5 target	0.288	0.183		0.268

Table 15: Partial correlation analysis of *NKX2-5* expression linking the SNP rs9481842 and TF activity.

Two-sided Pearson's correlation tests unless stated otherwise.

TF, transcription factor; *one-sided Pearson's correlation test;

Measure	SNP and *NKX2-5* mRNA	SNP and TF activity	*NKX2-5* mRNA and TF activity
Correlation	-0.43 ($P = 1.4\times10^{-4}$)	-0.13 (P = 0.13)*	0.36 ($P = 1.3\times10^{-4}$)*
Partial correlation	-0.41 ($P = 3\times10^{-4}$)	0.007 (P = 0.95)	0.3 (P = 0.011)
Condition	TF activity	*NKX2-5* mRNA	SNP

Table 16: NKX2-5 target correlations with *trans*-eQTL SNP rs9481842 and *NKX2-5* transcript as well as AF disease association.
All associations were computed by two-sided T tests based on a linear model with covariates (fibroblast-score, RIN-score or protein concentration). Correlation of SNP rs9481842 with target transcript (*trans*-eQTL) and target protein (*trans*-pQTL) were evaluated as well as the correlation between *NKX2-5* transcript expression and target protein expression. FDR based on the Benjamini-Hochberg procedure was only assessed for a preselected subset of genes when evaluating the correlation between the TF and target protein expression (see methods and Supplementary Fig. 13). Proteomics differential expression results are the same as reported in Table 4, were derived by two-sided T tests and additionally included common risk factors for AF as covariates (age, sex, BMI, diabetes, systolic blood pressure, hypertension medication, myocardial infarction and smoking) in the linear model.
SNP, single-nucleotide polymorphism; BS, number of binding sites; AF, atrial fibrillation; eQTL, expression quantitative trait loci; pQTL, protein quantitative trait loci; FDR, false discovery rate;
*Mutation known to affect cardiovascular phenotypes; **Mutation known to affect arrhythmias; $^{+}$Differential expression or functional impairment for cardiovascular phenotypes; $^{++}$Differential expression or functional impairment for arrhythmias; Source data are provided as a Source Data file.

NKX2-5 target		SNP rs9481842				*NKX2-5* target protein association			Disease association protein and AF	
		Trans-eQTL		*Trans*-pQTL						
Gene	BS	β	*P* value	β	*P* value	β	*P* value	FDR	β	*P* value
PPIF	1	-0.131	0.0081	-0.0388	0.0145	0.172	2.10×10^{-4}	0.00824	-0.0342	0.261
MYL4$^{**,++}$	1	-0.087	0.00921	-0.0359	0.0820	0.211	3.98×10^{-4}	0.00824	-0.0270	0.509
CKM^{++}	2	-0.115	0.0101	-0.0188	0.303	0.180	4.56×10^{-4}	0.00824	-0.0875	0.00705
MYL7	5	-0.125	0.0038	-0.0298	0.177	0.208	4.74×10^{-4}	0.00824	-0.0421	0.304
PGAM2^{++}	2	-0.214	0.00307	-0.0293	0.284	0.255	7.35×10^{-4}	0.0107	-0.175	0.000452
*TNNC1**	7	-0.0699	0.0359	-0.00809	0.623	0.147	1.69×10^{-3}	0.0211	-0.0557	0.0929
CYC1	3	-0.121	0.00278	-0.00651	0.749	0.175	3.16×10^{-3}	0.0307	-0.0946	0.0360
ETFB$^{**,++}$	2	-0.0924	0.0110	-0.0336	0.0563	0.152	3.39×10^{-3}	0.0307	-0.0553	0.105
PRDX5	6	-0.0710	0.00641	-0.0149	0.307	0.131	3.52×10^{-3}	0.0307	-0.0524	0.0789
AK1	3	-0.0654	0.0268	-0.0224	0.190	0.138	4.17×10^{-3}	0.0312	-0.0669	0.0341
ALDOA^{++}	7	-0.0555	0.0469	-0.00187	0.909	0.125	5.50×10^{-3}	0.0368	-0.0646	0.0341
*TCAP**	5	-0.114	0.00707	-0.0365	0.266	0.244	6.90×10^{-3}	0.0429	-0.0178	0.779
TOM1L2	2	-0.0886	0.0157	-0.0292	0.222	0.170	8.16×10^{-3}	0.0473	-0.0771	0.0849

Table 17: *Trans*-QTL results.
Significant *trans*- eQTLs and pQTLs at a FDR<0.2 (Benjamini-Hochberg procedure to account for multiple comparisons per omic). Two sided T tests were performed for 108 SNPs annotated with atrial fibrillation with 23 transcripts (N=74) and 152 proteins (N=73).
QTL, quantitative trait loci; eQTL, expression quantitative trait loci; pQTL, protein quantitative trait loci; FDR, false discovery rate;
*Mutation known to affect cardiovascular phenotypes; **Mutation known to affect arrhythmias; $^{+}$Differential expression or functional impairment for cardiovascular phenotypes; $^{++}$Differential expression or functional impairment for arrhythmias; Source data are provided as a Source Data file.

	Variant		Gene		*Trans*-eQTL				*Trans*-pQTL			
SNP	Chr	Position	Transcript	Chr	β	*T* value	*P* value	FDR	β	*T* value	*P* value	FDR
rs11658168	chr17	7,406,134	*TNNT2*$^{*,++}$	chr1	-0.517	-4.27	6.43×10^{-5}	**0.0812**	-0.213	-1.42	0.160	0.928
rs9481842	chr6	118,974,798	*NKX2-5***	chr5	-0.593	-4.27	6.54×10^{-5}	**0.0812**				
	**Variant**		**Gene**		***Trans*-eQTL**				***Trans*-pQTL**			
**SNP**	**Chr**	**Position**	**Protein**	**Chr**	β	***T* value**	***P* value**	**FDR**	β	***T* value**	***P* value**	**FDR**
rs11588763	chr1	154,813,584	CYB5R3	chr22	-0.119	-0.527	0.600	0.998	-0.786	-4.89	6.86×10^{-6}	**0.113**
rs11588763	chr1	154,813,584	NDUFB3^{+}	chr2	0.291	1.31	0.193	0.973	-0.916	-4.44	3.56×10^{-5}	**0.133**
rs11658168	chr17	7,406,134	HIBADH	chr7	-0.144	-0.861	0.393	0.997	-0.512	-4.43	3.66×10^{-5}	**0.133**
rs11588763	chr1	154,813,584	NDUFA9^{++}	chr12	0.257	1.16	0.249	0.985	-0.752	-4.42	3.85×10^{-5}	**0.133**
rs11588763	chr1	154,813,584	DLAT	chr11	0.470	2.56	0.0126	0.759	-0.716	-4.40	4.05×10^{-5}	**0.133**

Table 18: AFHRI cohort baseline table.
AF, atrial fibrillation; PRS, genome-wide polygenic score; BMI, body mass index; BP, blood pressure; conc., concentration;

Variable	All samples	eQTL/eQTS	pQTL/pQTS	AF association	All omics
Samples measured, n (%)	118 (100)	75 (64)	74 (63)	78 (66)	66 (56)
Women, n (%)	13 (11)	5 (7)	4 (5)	4 (5)	3 (5)
prevalent AF, n (%)	15 (13)	13 (17)	9 (12)	10 (13)	9 (14)
AF PRS, median (IQR)	32.40 (32.33-32.48)	32.40 (32.33-32.48)	32.40 (32.33-32.49)	32.39 (32.32-32.48)	32.39 (32.33-32.48)
Age, median (IQR), y	66.8 (59.5-73.5)	68.4 (60.6-73.8)	67.2 (59.7-73.7)	66.1 (58.6-72.0)	67.7 (60.6-73.7)
BMI, median (IQR), kg/m^2	27.8 (24.8-30.4)	28.3 (25.0-30.5)	27.6 (24.8-30.5)	26.9 (24.4-30.5)	28.0 (25.0-30.5)
systolic BP, median (IQR), mmHg	135 (122-145)	137 (123-146)	136 (122-145)	134 (122-145)	136 (123-145)
diastolic BP, median (IQR), mmHg	76 (70-82)	76 (70-81)	76 (70-80)	76 (70-80)	76 (70-80)
Hypertension, n (%)	105 (89)	68 (91)	66 (89)	68 (87)	60 (91)
Hypertension medication, n (%)	98 (83)	64 (85)	61 (82)	64 (82)	56 (85)
Diabetes, n (%)	36 (31)	25 (33)	23 (31)	25 (32)	22 (33)
Diabetes medication, n (%)	33 (28)	24 (32)	22 (30)	22 (28)	21 (32)
Myocardial infarction, n (%)	45 (38)	29 (39)	30 (41)	29 (37)	27 (41)
Smoking, n (%)	28 (24)	14 (19)	15 (20)	20 (26)	13 (20)
fibroblast-score, median (IQR)	80.43 (79.52-81.87)	80.42 (79.12-82.81)	80.26 (79.08-81.87)	80.34 (79.19-81.68)	80.26 (79.08-82.06)
RIN-score, median (IQR)	7.7 (7.1-8.1)	7.6 (7.1-8.1)	7.6 (7.1-8.1)	7.7 (7.3-8.1)	7.6 (7.1-8.1)
Protein conc., median (IQR), μg/μl	0.87 (0.45-1.31)	0.80 (0.45-1.30)	0.80 (0.45-1.32)	0.63 (0.43-1.32)	0.80 (0.45-1.30)

Table 19: ENCODE resources used to annotate RNA-binding proteins (RBP) binding sites. eCLIP data for all RBPs for Hep2 and K562 cell lines provided by Gene Yeo, UCSD.
`https://www.encodeproject.org/report/?type=Experiment&status=released&replicates.library.biosample.donor.organism.scientific_name=Homo+sapiens&biosample_ontology.classification=cell+line&assay_title=eCLIP&limit=all`

Accession	Assay	Target gene	Cell line	Accession	Assay	Target gene	Cell line	Accession	Assay	Target gene	Cell line
ENCSR999YGP	eCLIP	GRWD1	K562	ENCSR648LAH	eCLIP	DDX3X	HepG2	ENCSR001KKZ	eCLIP	PHF6	K562
ENCSR249ROI	eCLIP	HNRNPC	K562	ENCSR384MWO	eCLIP	CSTF2	HepG2	ENCSR721HPX	eCLIP	G3BP1	HepG2
ENCSR820UYE	eCLIP	PABPN1	HepG2	ENCSR154CSN	eCLIP	DDX52	K562	ENCSR464OSH	eCLIP	FUS	HepG2
ENCSR365NVO	eCLIP	TRA2A	K562	ENCSR331VNX	eCLIP	FMR1	K562	ENCSR906ZJF	eCLIP	SDAD1	K562
ENCSR337XGI	eCLIP	SAFB	HepG2	ENCSR987NYS	eCLIP	FAM120A	HepG2	ENCSR754NDA	eCLIP	RBM15	HepG2
ENCSR977OXG	eCLIP	PRPF4	HepG2	ENCSR861GYE	eCLIP	LIN28B	HepG2	ENCSR181NRW	eCLIP	ZC3H8	K562
ENCSR862QCH	eCLIP	U2AF1L5,U2AF1	K562	ENCSR970FEW	eCLIP	DDX52	HepG2	ENCSR322HHA	eCLIP	TIAL1	HepG2
ENCSR240MVJ	eCLIP	HNRNPU	HepG2	ENCSR973HOJ	eCLIP	FXR2	HepG2	ENCSR867ZVK	eCLIP	YWHAG	K562
ENCSR529FKI	eCLIP	YBX3	K562	ENCSR352STY	eCLIP	SSB	HepG2	ENCSR584TCR	eCLIP	TARDBP	K562
ENCSR841EQA	eCLIP	TAF15	HepG2	ENCSR050BDZ	eCLIP	SDAD1	HepG2	ENCSR999WKT	eCLIP	DDX24	K562
ENCSR022BVV	eCLIP	LSM11	K562	ENCSR685AUR	eCLIP	ZNF800	HepG2	ENCSR456KXI	eCLIP	LARP7	K562
ENCSR267OLV	eCLIP	SF3B4	K562	ENCSR089BXO	eCLIP	ABCF1	K562	ENCSR840DRD	eCLIP	CSTF2T	K562
ENCSR828ZID	eCLIP	HNRNPK	HepG2	ENCSR145NLR	eCLIP	DDX51	K562	ENCSR653HQC	eCLIP	DROSHA	K562
ENCSR406OOZ	eCLIP	SUB1	HepG2	ENCSR989VIY	eCLIP	SRSF1	HepG2	ENCSR331MIC	eCLIP	SF3A3	HepG2
ENCSR062NNB	eCLIP	IGF2BP2	K562	ENCSR194HZU	eCLIP	NOLC1	HepG2	ENCSR993OLA	eCLIP	IGF2BP3	HepG2
ENCSR267UCX	eCLIP	HNRNPM	HepG2	ENCSR979EWD	eCLIP	STAU2	HepG2	ENCSR006OEQ	eCLIP	FAM120A	K562
ENCSR196INN	eCLIP	RBM15	K562	ENCSR586DGV	eCLIP	ZNF800	K562	ENCSR755TJC	eCLIP	HNRNPUL1	HepG2
ENCSR571ROL	eCLIP	XRCC6	HepG2	ENCSR485QCG	eCLIP	BCCIP	HepG2	ENCSR773KRC	eCLIP	SRSF9	HepG2
ENCSR366YOG	eCLIP	QKI	K562	ENCSR279UJF	eCLIP	SF3B4	HepG2	ENCSR724RDN	eCLIP	HNRNPL	HepG2
ENCSR412NOW	eCLIP	HNRNPM	K562	ENCSR589YHM	eCLIP	HLTF	K562	ENCSR916SRV	eCLIP	TBRG4	HepG2
ENCSR135VMS	eCLIP	LSM11	HepG2	ENCSR893NWB	eCLIP	GRWD1	HepG2	ENCSR744GEU	eCLIP	IGF2BP1	HepG2
ENCSR661ICQ	eCLIP	PUM2	K562	ENCSR795CAI	eCLIP	HNRNPL	K562	ENCSR550DVK	eCLIP	HNRNPC	HepG2
ENCSR893EFU	eCLIP	DDX6	K562	ENCSR200DKE	eCLIP	MTPAP	K562	ENCSR543TPH	eCLIP	AGGF1	HepG2
ENCSR059CWF	eCLIP	SBDS	K562	ENCSR805SRN	eCLIP	LARP4	HepG2	ENCSR887FHF	eCLIP	FASTKD2	K562
ENCSR947JVR	eCLIP	DGCR8	K562	ENCSR845VGB	eCLIP	DDX55	HepG2	ENCSR819XBT	eCLIP	AATF	K562
ENCSR356ZMO	eCLIP	AKAP1	HepG2	ENCSR023UHL	eCLIP	FASTKD2	HepG2	ENCSR506UPY	eCLIP	SUGP2	HepG2
ENCSR922WJV	eCLIP	PCBP1	K562	ENCSR265ZIS	eCLIP	GTF2F1	HepG2	ENCSR489ABS	eCLIP	RBM5	HepG2
ENCSR975KIR	eCLIP	IGF2BP1	K562	ENCSR486YGP	eCLIP	FUBP3	HepG2	ENCSR351PVI	eCLIP	SLTM	HepG2
ENCSR177QQY	eCLIP	AKAP1	K562	ENCSR290VLT	eCLIP	MATR3	HepG2	ENCSR887LPK	eCLIP	EWSR1	K562
ENCSR766FAC	eCLIP	RPS3	HepG2	ENCSR993FMY	eCLIP	TROVE2	HepG2	ENCSR820DQJ	eCLIP	NOL12	HepG2
ENCSR539BEV	eCLIP	UPF1	HepG2	ENCSR295OKT	eCLIP	RBM22	K562	ENCSR981WKN	eCLIP	PTBP1	K562
ENCSR046JHH	eCLIP	CPEB4	K562	ENCSR769UEW	eCLIP	HNRNPA1	HepG2	ENCSR532VUB	eCLIP	CPSF6	K562
ENCSR023PKW	eCLIP	EIF3G	K562	ENCSR154HRN	eCLIP	HNRNPA1	K562	ENCSR061SZV	eCLIP	DGCR8	HepG2
ENCSR121GQH	eCLIP	SERBP1	K562	ENCSR830BSQ	eCLIP	BUD13	HepG2	ENCSR384KAN	eCLIP	PTBP1	HepG2
ENCSR366DGX	eCLIP	KHSRP	HepG2	ENCSR456JJQ	eCLIP	RBM22	HepG2	ENCSR958FKZ	eCLIP	PABPC4	K562
ENCSR141OIM	eCLIP	DDX6	HepG2	ENCSR238CLX	eCLIP	GEMIN5	K562	ENCSR739VVT	eCLIP	APOBEC3C	K562
ENCSR520BZQ	eCLIP	HNRNPU	K562	ENCSR438KWZ	eCLIP	ILF3	K562	ENCSR038JME	eCLIP	WRN	K562
ENCSR432XUP	eCLIP	SRSF1	K562	ENCSR488JKQ	eCLIP	UTP18	HepG2	ENCSR921SXC	eCLIP	XPO5	HepG2
ENCSR534YOI	eCLIP	PRPF8	K562	ENCSR018WPY	eCLIP	AQR	HepG2	ENCSR085JPB	eCLIP	WDR43	HepG2
ENCSR529GSJ	eCLIP	DHX30	K562	ENCSR964VOX	eCLIP	UTP18	K562	ENCSR734ZHL	eCLIP	UTP3	K562
ENCSR373ODC	eCLIP	SMNDC1	HepG2	ENCSR655NZA	eCLIP	XRN2	HepG2	ENCSR815VVI	eCLIP	CDC40	HepG2
ENCSR867DSZ	eCLIP	NPM1	K562	ENCSR725ARB	eCLIP	AGGF1	K562	ENCSR061EVO	eCLIP	SND1	HepG2
ENCSR834YLD	eCLIP	DROSHA	HepG2	ENCSR668MJX	eCLIP	GRSF1	HepG2	ENCSR121NVA	eCLIP	PRPF8	HepG2
ENCSR268ETU	eCLIP	HNRNPK	K562	ENCSR570WLM	eCLIP	QKI	HepG2	ENCSR844RVX	eCLIP	EFTUD2	K562
ENCSR081JYH	eCLIP	NSUN2	K562	ENCSR018ZUE	eCLIP	FKBP4	HepG2	ENCSR506OTC	eCLIP	TBRG4	K562
ENCSR339FUY	eCLIP	PCBP2	HepG2	ENCSR269AJF	eCLIP	RPS11	K562	ENCSR197INS	eCLIP	PPIL4	K562
ENCSR923NKN	eCLIP	DDX55	K562	ENCSR440SUX	eCLIP	MATR3	K562	ENCSR861PAR	eCLIP	NONO	K562
ENCSR970NKP	eCLIP	LIN28B	K562	ENCSR903PRV	eCLIP	FTO	HepG2	ENCSR987FTF	eCLIP	RBFOX2	HepG2
ENCSR301UQM	eCLIP	GNL3	K562	ENCSR256CHX	eCLIP	PCBP1	HepG2	ENCSR224QWC	eCLIP	FXR2	K562
ENCSR484LTQ	eCLIP	NCBP2	K562	ENCSR961OKA	eCLIP	LARP7	HepG2	ENCSR663WES	eCLIP	BUD13	K562
ENCSR888YTT	eCLIP	LARP4	K562	ENCSR565DGW	eCLIP	DHX30	HepG2	ENCSR303OQD	eCLIP	METAP2	K562
ENCSR018RVZ	eCLIP	NCBP2	HepG2	ENCSR120EAR	eCLIP	RPS3	K562	ENCSR206RXT	eCLIP	AKAP8L	K562
ENCSR097NEE	eCLIP	PPIG	HepG2	ENCSR663NRA	eCLIP	ZRANB2	K562	ENCSR893RAV	eCLIP	U2AF2	K562
ENCSR930BZL	eCLIP	DDX3X	K562	ENCSR786TSC	eCLIP	ILF3	HepG2	ENCSR041NUV	eCLIP	EIF3D	HepG2
ENCSR965DLL	eCLIP	SFPQ	HepG2	ENCSR001VAC	eCLIP	NOLC1	K562	ENCSR657TZB	eCLIP	XRN2	K562
ENCSR277DEO	eCLIP	NKRF	HepG2	ENCSR308YNT	eCLIP	PUM1	K562	ENCSR916XIV	eCLIP	EIF3H	HepG2
ENCSR202BFN	eCLIP	U2AF2	HepG2	ENCSR128VXC	eCLIP	SND1	K562	ENCSR736AAG	eCLIP	GTF2F1	K562
ENCSR202HKN	eCLIP	WDR3	K562	ENCSR647HOX	eCLIP	HLTF	HepG2	ENCSR647CLF	eCLIP	GPKOW	K562
ENCSR774RFN	eCLIP	FXR1	K562	ENCSR606BPV	eCLIP	AQR	K562	ENCSR301TFY	eCLIP	DKC1	HepG2
ENCSR658IQB	eCLIP	SMNDC1	K562	ENCSR876EYA	eCLIP	BCLAF1	HepG2	ENCSR214BZA	eCLIP	DDX59	HepG2
ENCSR349CMI	eCLIP	WDR43	K562	ENCSR438GZQ	eCLIP	KHSRP	K562	ENCSR820WHR	eCLIP	POLR2G	HepG2
ENCSR040QLV	eCLIP	DDX21	K562	ENCSR756CKJ	eCLIP	RBFOX2	K562	ENCSR490IEE	eCLIP	UCHL5	HepG2
ENCSR891RIC	eCLIP	NIPBL	K562	ENCSR693JWP	eCLIP	EXOSC5	HepG2	ENCSR291XPT	eCLIP	PUS1	K562
ENCSR356MSW	eCLIP	SSB	K562	ENCSR307YIW	eCLIP	EIF4G2	K562	ENCSR943MHU	eCLIP	SAFB2	K562
ENCSR735HOK	eCLIP	YBX3	HepG2	ENCSR349KMG	eCLIP	UCHL5	K562	ENCSR314UMJ	eCLIP	TRA2A	HepG2
ENCSR571VHI	eCLIP	HNRNPUL1	K562	ENCSR712IAG	eCLIP	ZC3H11A	K562	ENCSR328LLU	eCLIP	U2AF1L5,U2AF1	HepG2
ENCSR580MFX	eCLIP	SUPV3L1	HepG2	ENCSR361OCV	eCLIP	NIP7	HepG2	ENCSR989SMC	eCLIP	FTO	K562
ENCSR527DXF	eCLIP	EFTUD2	HepG2	ENCSR623VEQ	eCLIP	TIA1	HepG2	ENCSR907GUB	eCLIP	ZC3H11A	HepG2
ENCSR468FSW	eCLIP	SRSF7	K562	ENCSR069EVH	eCLIP	FUS	K562	ENCSR628IDK	eCLIP	KHDRBS1	K562
ENCSR483NOP	eCLIP	SLBP	K562	ENCSR057DWB	eCLIP	TIA1	K562	ENCSR539ZTS	eCLIP	TROVE2	K562
ENCSR000SSH	eCLIP	SLTM	K562	ENCSR580OFI	eCLIP	SUPV3L1	K562	ENCSR133QEA	eCLIP	SF3B1	K562
ENCSR919HSE	eCLIP	CSTF2T	HepG2	ENCSR657TZZ	eCLIP	ZNF622	K562	ENCSR013CTQ	eCLIP	EXOSC5	K562
ENCSR576SHT	eCLIP	DDX42	K562	ENCSR568DZW	eCLIP	TAF15	K562	ENCSR456ASB	eCLIP	UPF1	K562
ENCSR513NDD	eCLIP	SRSF7	HepG2	ENCSR258QKO	eCLIP	XRCC6	K562				
ENCSR825SVO	eCLIP	AARS	K562	ENCSR484LAB	eCLIP	SAFB	K562				

Supplementary Information

Supplementary Note 1

Cis-QTL replication

We compared QTLs across omics for our AFHRI cohort, across studies to the GTEx[1] atrial appendage tissue and across tissues to plasma *cis*-pQTLs. QTLs replicated well across omics and studies to the GTEx data, however significance levels varied strongly when compared to plasma pQTL data.
We systematically compared our AFHRI eQTLs to eQTLs for right atrial appendage tissue of the GTEx project. For the comparison, we first filtered the GTEx data for our measured SNPs and genes. We selected the most significant marker for each gene with *cis*-eQTL in our study (P <$1{\times}10^{-5}$) and compared allelic effects. Of all genes available for replication (54%), 62% replicated, 87% showed concordant effects (25% showed concordant effects without significance), and none of the eQTLs significant in both datasets showed discordant effects. Using Storey's q-value method[20], we estimated a replication rate of 85%.
Conversely, of the top GTEx SNPs analyzed in our study, 85% showed concordant allelic effects and 7.8% replicated. The low replication rate is probably due to the large difference in sample size, indicated by the high rate of concordant allelic effects.

Correlation between mRNA and protein

Protein and transcript levels showed a median correlation of 0.15 per gene and 0.21 per sample. Fitting a linear model to explain protein abundance from transcript expression showed a median R^2 of 0.027 per gene and 0.044 per sample (N=79). This general modest mRNA-protein correlation was observed by other comparable datasets as well (Supplementary Table 1)[2]. Correlations did not increase much if considering only genes with a *cis*-eQTL, however, highly variable genes showed a median correlation of 0.23.
To evaluate the variability of a gene, we compared the logarithm of the variance per gene (over all samples) σ_G to the median expression m_G and fit the linear model $\log(\sigma_G) \sim \beta_0 + \beta_1 \cdot m_G + \varepsilon$. Highly variable genes were then defined as all genes i with $\sigma_{G_i} > \exp(\hat{\beta}_0 + \hat{\beta}_1 \cdot m_G + 3 \cdot \hat{\sigma}(\hat{\beta}_1))$.

Overlap of *cis*-eQTLs and pQTLs:

We used Storey's qvalue method[20] to estimate replication rates between the top significant *cis*-QTLs in transcriptomics and proteomics. For a FDR $<$ 0.05 significance cutoff, 32% of the top eQTLs replicated in pQTLs and 50% of the top pQTLs in eQTLs. We observe a Pearson correlation of 0.58 for all significant eQTL SNP-gene pairs and their corresponding pQTLs and a correlation of 0.75 for the effect sizes of all significant pQTL SNP-gene pairs when compared to the matching eQTLs. Similarly, for a P value cutoff $P < 1 \times 10^{-5}$, replication rates of 20%/32% for the top QTL per gene and effect size correlations of 0.61 and 0.79 for all SNP-gene pairs were determined.
Previous studies reported an overlap of 40% of plasma pQTLs[3] with a corresponding eQTL from the GTEx study[1]. However, these eQTLs were not specific for plasma but arose from all available GTEx tissues. Restricting to eQTLs found in whole blood, 19% of plasma pQTLs had a corresponding eQTL. Based on supplemental information given by the authors, the fraction of eQTLs in heart tissues (atrial appendage and left ventricle) that overlapped with plasma pQTLs was much smaller (<7.5%). The other way around, depending on the tissue-specific eQTLs, 12% to 21% of eQTLs also had a plasma pQTL which is well within the range of 17% we observed in our dataset. That implies a qualitatively comparable and reliable overlap between pQTLs and eQTLs which probably is even underestimated. For instance, when using a less stringent significance cutoff of FDR<0.1, a much higher coverage of measured proteins (4,381) and restricting the multiple testing burden by

using less SNP-gene pairs in the discovery dataset, Battle and colleagues achieved a much higher replication rate[2]. For matched transcriptomics and proteomics measurements in lymphoblastoid cell lines (LCL), they replicated 35% of eQTL SNP-gene pairs on proteomics level, compared to 14% in our study and 67% of pQTL SNP-gene pairs showed an eQTL, which was significantly more than 16% in our study.
It has to be mentioned, that the way of comparing QTLs differed vastly across studies. Battle and colleagues used overlapping SNP-gene pairs, without taking linkage information into account[2]. Sun and colleagues first defined lead SNPs in high LD regions ($r^2 \geq 0.8$) for the same gene[3]. *Cis*-pQTLs in LCLs were also evaluated by Hause and colleagues[4] who found no overlapping *cis*-pQTLs and *cis*-eQTLs at their original significance threshold of FDR<0.05.

Enrichment of functional elements

To further elucidate regulatory mechanisms for the identified QTL associations, we annotated each variant-gene pair with publicly available functional genomics annotations, such as position in the exon, 5' UTR, 3' UTR or splice sites, microRNA binding sites (miRNA BS), transcription factor binding sites (TF BS), RNA-binding protein binding sites (RBP BS), chromatin states, possible missense mutations or nonsense mediated decay (NMD) (Supplementary Fig. 7).
Amongst others, there was a significant enrichment (P<0.05, two-sided Fisher's exact test) of active transcription start sites (TssA), enhancer, exon, splice, 5' UTR and 3' UTR regions in eQTLs. Similar results have been reported by Lappalainen and colleagues[21].
Furthermore, independent pQTLs were enriched in exons, which was previously reported by Battle and colleagues for the comparable group of protein specific QTLs[2].

GWAS catalog overlaps

Due to the strong signal of AF in our QTL results, we further investigated specific hits. We replicated the strong associations at the SYNPO2L locus, where genetic variation strongly influences MYOZ1 but not SYNPO2L. Only 2 (MYOZ1, PCCB) out of the 7 genes with eQTLs that overlapped with AF GWAS hits were measured on proteomics level. The MYOZ1 locus showed a strong eQTL and pQTL, where the QTL and GWAS P values[22] were highly correlated.

Supplementary References

1. Gamazon, E. R. *et al.* Using an Atlas of Gene Regulation across 44 Human Tissues to Inform Complex Disease- and Trait-Associated Variation. *Nature Genetics* **50,** 956–967 (2018).
2. Battle, A. *et al.* Impact of Regulatory Variation from RNA to Protein. *Science* **347,** 664–667 (2015).
3. Sun, B. B. *et al.* Genomic Atlas of the Human Plasma Proteome. *Nature* **558,** 73–79 (2018).
4. Hause, R. J. *et al.* Identification and Validation of Genetic Variants That Influence Transcription Factor and Cell Signaling Protein Levels. *Am. J. Hum. Genet.* **95,** 194–208 (2014).
5. Wang, B. *et al.* Integrative Omics Approach to Identifying Genes Associated With Atrial Fibrillation. *Circ. Res.* **126,** 350–360 (2020).
6. Amberger, J. S., Bocchini, C. A., Schiettecatte, F., Scott, A. F. & Hamosh, A. OMIM.Org: Online Mendelian Inheritance in Man (OMIM®), an Online Catalog of Human Genes and Genetic Disorders. *Nucleic Acids Res.* **43,** D789–D798 (2015).
7. Hershberger, R. E. *et al.* Clinical and Functional Characterization of TNNT2 Mutations Identified in Patients with Dilated Cardiomyopathy. *Circ. Cardiovasc. Genet.* **2,** 306–313 (2009).

8. Doll, S. *et al.* Region and Cell-Type Resolved Quantitative Proteomic Map of the Human Heart. *Nat. Commun.* **8,** 1469 (2017).
9. Jhaveri, S., Aziz, P. F. & Saarel, E. Expanding the Electrical Phenotype of NKX2-5 Mutations: Ventricular Tachycardia, Atrial Fibrillation, and Complete Heart Block within One Family. *Hear. Case Rep.* **4,** 530–533 (2018).
10. Huang, R. T., Xue, S., Xu, Y. J., Zhou, M. & Yang, Y. Q. A Novel NKX2.5 Loss-of-Function Mutation Responsible for Familial Atrial Fibrillation. *Int. J. Mol. Med.* **31,** 1119–1126 (2013).
11. Kanaan, G. N., Patten, D. A., Redpath, C. J. & Harper, M. E. Atrial Fibrillation Is Associated With Impaired Atrial Mitochondrial Energetics and Supercomplex Formation in Adults With Type 2 Diabetes. *Can. J. Diabetes* **43,** 67–75 (2019).
12. El-Hattab, A. W. & Scaglia, F. Mitochondrial Cardiomyopathies. *Front. Cardiovasc. Med.* **3,** 25 (2016).
13. Orr, N. *et al.* A Mutation in the Atrial-Specific Myosin Light Chain Gene (MYL4) Causes Familial Atrial Fibrillation. *Nat. Commun.* **7,** 11303 (2016).
14. Tu, T., Zhou, S., Liu, Z., Li, X. & Liu, Q. Quantitative Proteomics of Changes in Energy Metabolism-Related Proteins in Atrial Tissue from Valvular Disease Patients with Permanent Atrial Fibrillation. *Circ. J.* **78,** 993–1001 (2014).
15. Coats, C. J. *et al.* Proteomic Analysis of the Myocardium in Hypertrophic Obstructive Cardiomyopathy. *Circ. Genomic Precis. Med.* **11,** e001974 (2018).
16. Parvatiyar, M. S. *et al.* A Mutation in TNNC1-Encoded Cardiac Troponin C, TNNC1-A31S, Predisposes to Hypertrophic Cardiomyopathy and Ventricular Fibrillation. *J. Biol. Chem.* **287,** 31845–31855 (2012).
17. Florian, A. R. & Yilmaz, A. in *Diagnosis and Management of Mitochondrial Disorders* (Springer, Basel, 2019).
18. Hayashi, T. *et al.* Tcap Gene Mutations in Hypertrophic Cardiomyopathy and Dilated Cardiomyopathy. *J. Am. Coll. Cardiol.* **44,** 2192–2201 (2004).
19. Dalal, R. S., Sabe, A. A., Elmadhun, N. Y., Ramlawi, B. & Sellke, F. W. Atrial Fibrillation, Neurocognitive Decline and Gene Expression after Cardiopulmonary Bypass. *Rev. Bras. Cir. Cardiovasc.* **30,** 520–532 (2015).
20. Storey, J. D. & Tibshirani, R. Statistical Significance for Genomewide Studies. *Proc. Natl. Acad. Sci.* **100,** 9440–9445 (2003).
21. Lappalainen, T. *et al.* Transcriptome and Genome Sequencing Uncovers Functional Variation in Humans. *Nature* **501,** 506–511 (2013).
22. Roselli, C. *et al.* Multi-Ethnic Genome-Wide Association Study for Atrial Fibrillation. *Nat. Genet.* **50,** 1225–1233 (2018).
