# Supplementary material for: Tissue-specific multi-omics analysis of atrial fibrillation
Source: Nat Commun. 2022 Jan 21;13:441. doi: 10.1038/s41467-022-27953-1 (PMC8782899; doi:10.1038/s41467-022-27953-1)
Supplement: Supplementary file 3 — Reporting Summary [file 41467_2022_27953_MOESM3_ESM.pdf]

nature research

Corresponding author(s): Matthias Heinig
Renate Schnabel

Last updated by author(s): Aug 17, 2020

Reporting Summary

Nature Research wishes to improve the reproducibility of the work that we publish. This form provides structure for consistency and transparency in reporting. For further information on Nature Research policies, see our Editorial Policies and the Editorial Policy Checklist.

Statistics

For all statistical analyses, confirm that the following items are present in the figure legend, table legend, main text, or Methods section.

n/a	Confirmed	
☐	☒	The exact sample size (n) for each experimental group/condition, given as a discrete number and unit of measurement
☐	☒	A statement on whether measurements were taken from distinct samples or whether the same sample was measured repeatedly
☐	☒	The statistical test(s) used AND whether they are one- or two-sided<br>*Only common tests should be described solely by name; describe more complex techniques in the Methods section.*
☐	☒	A description of all covariates tested
☐	☒	A description of any assumptions or corrections, such as tests of normality and adjustment for multiple comparisons
☐	☒	A full description of the statistical parameters including central tendency (e.g. means) or other basic estimates (e.g. regression coefficient) AND variation (e.g. standard deviation) or associated estimates of uncertainty (e.g. confidence intervals)
☐	☒	For null hypothesis testing, the test statistic (e.g. F, t, r) with confidence intervals, effect sizes, degrees of freedom and P value noted<br>*Give P values as exact values whenever suitable.*
☒	☐	For Bayesian analysis, information on the choice of priors and Markov chain Monte Carlo settings
☒	☐	For hierarchical and complex designs, identification of the appropriate level for tests and full reporting of outcomes
☐	☒	Estimates of effect sizes (e.g. Cohen's d, Pearson's r), indicating how they were calculated

Our web collection on statistics for biologists contains articles on many of the points above.

Software and code

Policy information about availability of computer code

Data collection	Rosetta elucidator 3.3 was used to collect proteomics data.<br>Affymetrix GeneChip 3000 scanner with the Affymetrix Genechip Command Console 4.0.0.1567 were used to collect expression and genotyping microarray data.<br>The Western Blots were analysed using the Bio-Rad software (Image Lab 6.1).
Data analysis	Data analysis was performed with R 3.4.1, R 3.6.3 (peer 1.0, MatrixEQTL 2.2, impute 1.50.1, fgsea 1.8.0/1.12.0, rtracklayer 1.46.0, GenomicRanges 1.38.0, coloc 3.2-1, ppcor 1.1, circlize 0.4.8), Birdseed v2, IMPUTE2 (2.3.2), Plink 1.9. In addition, custom code is available on github https://github.com/heiniglab/symatrial [https://doi. org/10.5281/zenodo.5094276]

For manuscripts utilizing custom algorithms or software that are central to the research but not yet described in published literature, software must be made available to editors and reviewers. We strongly encourage code deposition in a community repository (e.g. GitHub). See the Nature Research guidelines for submitting code & software for further information.

Data

Policy information about availability of data

All manuscripts must include a data availability statement. This statement should provide the following information, where applicable:

- Accession codes, unique identifiers, or web links for publicly available datasets
- A list of figures that have associated raw data
- A description of any restrictions on data availability

The genotype, transcriptomics and proteomics data are available under restricted access, as they contain identifying participant information. Deposition in online repositories or controlled access repositories is not mandated by the patient's consent. Access can be obtained by qualified researchers upon reasonable request to the corresponding authors. Mandated source data are provided with this paper. All results are available at http://qtldb.helmholtz-muenchen.de and in the Zenodo

repository [https://doi.org/10.5281/zenodo.5080229][83]. For replication purposes, publicly available data were obtained from GTEx [https://www.gtexportal.org/home/datasets] (RNA-seq and cis-eQTL results), GSE128188 [https://www.ncbi.nlm.nih.gov/geo/query/acc.cgi?acc=GSE128188] and PXD006675 [https://www.ebi.ac.uk/pride/archive/projects/PXD006675]. Additionally, the following annotations were used Ensembl BioMart GRCh37.p13: hg19 and Ensembl Variant Effect Predictions [http://feb2014.archive.ensembl.org/biomart/martview/], 1000 Genomes Phase 3 genotypes [https://www.internationalgenome.org/], GWAS catalog [https://www.ebi.ac.uk/gwas/] (2019-11-26), Roadmap Epigenomics E104_15_coreMarks_dense.bed [https://egg2.wustl.edu/roadmap/data/byFileType/chromhmmSegmentations/ChmmModels/coreMarks/jointModel/final/], E-MTAB-6014 capt-CM-replicated-interactions-1kb.bedpe [https://www.ebi.ac.uk/arrayexpress/experiments/E-MTAB-6014/], ReMap2018 v1.2 [http://pedagogix-tagc.univ-mrs.fr/remap/download/remap2018/hg19/MACS/remap2018_nr_macs2_hg19_v1_2.bed.gz], TargetScan 7.2 [http://www.targetscan.org/vert_72/vert_72_data_download/Predicted_Target_Locations.default_predictions.hg19.bed.zip], GSE133833 [https://www.ncbi.nlm.nih.gov/geo/query/acc.cgi?acc=GSE133833] and ENCODE eCLIP HepG2/K562 data [https://encodeproject.org].

Field-specific reporting

Please select the one below that is the best fit for your research. If you are not sure, read the appropriate sections before making your selection.

- [x] Life sciences
- [ ] Behavioural & social sciences
- [ ] Ecological, evolutionary & environmental sciences

For a reference copy of the document with all sections, see nature.com/documents/nr-reporting-summary-flat.pdf

Life sciences study design

All studies must disclose on these points even when the disclosure is negative.

Sample size	Analyses were exploratory in nature. Therefore No sample size calculation was performed. Sample size was limited by the limited availability of human heart tissue samples. Prior cis-eQTL studies with similar sample sizes in the literature showed that power for detecting strong cis-eQTL is sufficiently high (GTEx sample size vs number of eQTL, PMID: 29022597). Most other multi-omics studies to date have relied on similar sample sizes, in particular, when cardiac tissue samples were involved (PMID: 29555953 [N = 60], 30562113 [N = 65]).
Data exclusions	Samples (genotyping, gene expression microarrays and proteomics measurements) were excluded from the QTL or integrated analyses based on availability of biomaterial and data quality control criteria defined in the methods section.
Replication	Cis eQTL findings were succesfully replicated in atrial tissue from GTEx. No replication data set with proteomics QTLs in atrial tissue was available. Differential expression of core gene candidates was replicated in previously published RNA-seq (GSE128188) and proteomics (PXD006675) experiments. Western blot analysis was done using biological replicates of individuals with different genotypes of rs9481842 (n(TT) = 14, n(TG) = 11, n(GG) = 4) and no technical replicates were used. No additional replication experiments were carried out.
Randomization	We report on an observational study, therefore no experimental groups were assigned. Potential confounding factors were included in the analyses as covariates.
Blinding	Investigators were blinded to diseases status during the data collection. For QTL analysis blinding is not relevant as the grouping of samples is based on genotypes and changes for each SNP. For the analysis of the association of omics and AF phenotype blinding is not possible.

Reporting for specific materials, systems and methods

We require information from authors about some types of materials, experimental systems and methods used in many studies. Here, indicate whether each material, system or method listed is relevant to your study. If you are not sure if a list item applies to your research, read the appropriate section before selecting a response.

Materials & experimental systems

n/a	Involved in the study	
[ ]	[x]	Antibodies
[x]	[ ]	Eukaryotic cell lines
[x]	[ ]	Palaeontology and archaeology
[x]	[ ]	Animals and other organisms
[ ]	[x]	Human research participants
[x]	[ ]	Clinical data
[x]	[ ]	Dual use research of concern

Methods

n/a	Involved in the study	
[x]	[ ]	ChIP-seq
[x]	[ ]	Flow cytometry
[x]	[ ]	MRI-based neuroimaging

Antibodies

Antibodies used	NKX2-5 (ab205263, clone: EPR20168, Lot: GR3198814/2)<br>alpha actinin (CST #3134, Lot: 2/2020)<br>GAPDH (CST #3683, Lot: 4/2020)

Validation	Manufacturers provide validation statements of the antibodies for Western blot applications in human tissues on their websites. For all antibodies, molecular masses observed are consistent with what has been described in the literature.

Human research participants

Policy information about studies involving human research participants

Population characteristics	The population includes 118 patients undergoing coronary bypass surgery. The mean age was 66.8 years (IQR: 59.5-73.5). Most participants were male (105: 89%) and all were of european ancestry. All patients were diagnosed with coronary artery disease and 15 (13%) were diagnosed with prevalent atrial fibrillation. In addition 105 (89%) patients were diagnosed with hypertension and 36 (31%) with diabetes. All patients underwent coronary bypass surgery, 98 (89%) were treated with hypertension medication and 33 (28%) with diabetes medication.
Recruitment	Patients were consecutively enrolled in the ongoing observational cohort study AFHRI-B (Atrial fibrillation in high risk individuals-biopsy) independent of AF disease status. Participants were older than 18 years of age and were scheduled to undergo open heart coronary bypass surgery. Patients with surgery other than coronary bypass, e.g. valve surgery, were excluded. Participation in the observational study was voluntary and may have led to self-selection bias. However, the consecutive enrolment of patients was implemented in order to reduce bias. The overall cohort largely resembled a stable cohort of patients undergoing CABG.
Ethics oversight	The observational cohort study was approved by the Ethikkommission Ärztekammer Hamburg (PV3982).

Note that full information on the approval of the study protocol must also be provided in the manuscript.
